# Peripheral blood cell counts as predictors of immune-related adverse events in cancer patients receiving immune checkpoint inhibitors: a systematic review and meta-analysis

**DOI:** 10.3389/fimmu.2025.1528084

**Published:** 2025-01-30

**Authors:** Xinyu Zhang, Bei Zhang, Danfei Li, Yunchao Yang, Sen Lin, Ruiqi Zhao, Yijia Li, Lisheng Peng

**Affiliations:** ^1^ The Fourth Clinical Medical College of Guangzhou University of Chinese Medicine, Shenzhen, Guangdong, China; ^2^ Shandong College of Traditional Chinese Medicine, Shandong, Yantai, China; ^3^ Department of Hepatology, Shenzhen Traditional Chinese Medicine Hospital, Shenzhen, China

**Keywords:** immune checkpoint inhibitors, immunotherapy, immune-related adverse events, blood cell count, biomarker, risk factor

## Abstract

**Background:**

In recent years, immune checkpoint inhibitors (ICIs) have shown significant efficacy in treating various malignancies and have become a key therapeutic approach in cancer treatment. However, while ICIs activate the immune system, they can also induce immune-related adverse events (irAEs). Due to the variability in the frequency and severity of irAEs, clinical management faces a significant challenge in balancing antitumor efficacy with the risk of irAEs. Predicting and preventing irAEs during the early stages of treatment has become a critical research focus in cancer immunotherapy. This study aims to evaluate the predictive value of peripheral blood cell counts for irAEs.

**Methods:**

Studies meeting the inclusion criteria were identified through database searches. The standardized mean difference (SMD) was used to compare continuous blood cell counts. For studies that did not provide adjusted odds ratios (ORs) and 95% confidence intervals (CIs), crude ORs for categorized blood cell counts were calculated. The study protocol was registered on PROSPERO (CRD42024592126).

**Results:**

The meta-analysis included 60 studies involving 16,736 cancer patients treated with ICIs. Compared to patients without irAEs, those experiencing irAEs had significantly higher baseline continuous ALC (SMD = 0.12, 95% CI = 0.01-0.24), while ANC (SMD = -0.18, 95% CI = -0.28 to -0.07) and PLR (SMD = -0.32, 95% CI = -0.60 to -0.04) were significantly lower. Similarly, categorized blood cell counts indicated that higher baseline ALC (OR = 2.46, 95% CI = 1.69-3.57) and AEC (OR = 2.05, 95% CI = 1.09-3.85), along with lower baseline NLR (OR = 0.64, 95% CI = 0.50-0.81) and PLR (OR = 0.63, 95% CI = 0.48-0.82), were associated with an increased risk of irAEs. Subgroup analysis further identified cutoff values for ALC (2×10^9/L), NLR (5 or 3), and PLR (180) as better predictors of irAEs.

**Conclusion:**

Higher baseline ALC and AEC, along with lower baseline ANC, NLR, and PLR, are associated with an increased risk of irAEs. However, further research is needed to determine the optimal cutoff values and to explore the efficacy of blood cell counts in predicting specific types of irAEs.

**Systematic review registration:**

https://www.crd.york.ac.uk/PROSPERO/, identifier CRD42024592126.

## Introduction

Cancer immunotherapy has emerged as a breakthrough in the treatment of various malignancies. ICIs, which target PD-1/PD-L1 and CTLA-4 pathways, work by blocking inhibitory signals, activating T cells, and reinvigorating antitumor immune responses. However, by enhancing host immune responses and disrupting immune homeostasis, ICIs can promote inflammatory activity, potentially leading to inflammation-related damage in multiple organs (1). This manifests as a range of clinical symptoms collectively called irAEs, commonly affecting various organ systems, including the skin, endocrine, respiratory, and gastrointestinal systems (2). The incidence of irAEs is relatively high, and certain severe complications can significantly affect patients’ quality of life and prognosis (3). Effectively managing irAEs without compromising the antitumor efficacy of ICIs or the long-term survival of patients remains a clinical challenge (4). Notably, patients who develop irAEs often experience better cancer outcomes (5–7). Therefore, assessing individual risk for toxicity in advance is crucial, as early intervention and management of irAEs can help ensure that high-risk patients continue ICI treatment and benefit from it.

As the use of ICIs in cancer treatment continues to expand, there is an increasing need for reliable and validated biomarkers to predict irAEs (8). Factors such as drug selection, gender, laboratory indicators, pre-existing comorbidities, and tumor mutation burden (TMB) have been identified as potential predictors of irAEs (9–11). However, these factors are often difficult to apply widely in clinical practice due to limited accuracy or high testing costs. Easily measurable and cost-effective markers like blood cell counts have garnered increasing attention. Circulating blood cell counts—such as absolute lymphocyte count (ALC), neutrophil to lymphocyte ratio (NLR), lymphocyte to monocyte ratio (LMR), and platelet to lymphocyte ratio (PLR)—have shown potential for predicting both the efficacy of cancer immunotherapy and the risk of irAEs (11–15). However, current research on the relationship between blood cell counts and irAEs has yielded inconsistent results, with many studies limited by small sample sizes or single-center analyses and lacking systematic reviews and quantitative assessments.

Through a systematic review and meta-analysis, this study aims to evaluate the predictive value of peripheral blood cell counts for irAEs in cancer patients receiving ICIs. Additionally, we seek to identify clinically relevant cutoffs for these blood cell counts, providing evidence-based support for clinical practice.

## Materials and methods

This study was conducted in accordance with the Preferred Reporting Items for Systematic Reviews and Meta-Analyses (PRISMA) 2020 guidelines. The study protocol was registered on PROSPERO (CRD42024592126). The objective was to assess the predictive value of peripheral blood cell counts for irAEs in cancer patients receiving ICIs.

### Search strategy

We conducted a comprehensive literature search in PubMed, Ovid Medline, Embase, and Cochrane Library databases, with a search cutoff date of August 24, 2024. The search terms included “immune checkpoint inhibitor”, “immune-related adverse events”, “neutrophils”, “lymphocytes”, “monocytes”, “eosinophils”, “platelets”, “neutrophil to lymphocyte ratio”, “platelet to lymphocyte ratio”, “monocyte to lymphocyte ratio”, “lymphocyte to monocyte ratio”, “risk factors”. The detailed search strategy is available in **Supplementary Table 1**.

### Inclusion and exclusion criteria

The inclusion criteria were established as follows (1): Studies included patients with cancer treated with ICIs; (2) The incidence of irAEs was reported; (3) The study evaluated blood cell counts as a predictive factor for irAEs; (4) The study was a randomized clinical trial, retrospective clinical study, or case-control study.

The exclusion criteria were established as follows: (1) Studies involving *in vitro* or *in vivo* experiments. (2) Lack of available data on continuous blood cell counts, categorized blood cell counts by cut-off, or ORs associated with irAEs. (3) Case reports or case series with a sample size of less than 10.

### Literature screening, data extraction

Two researchers independently screened the titles and abstracts based on the inclusion and exclusion criteria. The full texts were further evaluated if the abstracts lacked sufficient detail or the data could not be extracted. Any disagreements between the two reviewers were resolved through discussion with a third investigator. Data from the eligible studies were extracted into a standardized form, including study characteristics (e.g., author, year, design), patient characteristics (e.g., age, sex, cancer type), blood cell count-related variables (including absolute neutrophil count (ANC), absolute lymphocyte count (ALC), absolute monocyte count (AMC), absolute eosinophil count (AEC), platelet count (PLT), neutrophil to lymphocyte ratio (NLR), platelet to lymphocyte ratio (PLR) and monocyte to lymphocyte ratio (MLR)), as well as the incidence of irAEs either overall or by specific subtypes. These blood cell counts and percentages were recorded at baseline, before the initiation of ICI therapy. The continuous or categorized values of these blood cell counts were collected in terms of adverse event (AE) and non-AE groups. ORs with corresponding 95% CIs were also collected when available. Multivariate or adjusted ORs were preferentially included; otherwise, univariate ORs were included or calculated based on the original data from the article.

### Quality assessment

The quality of the included studies was assessed using the Newcastle-Ottawa Scale (NOS) tool. Any discrepancies were resolved through the involvement of a third party until a consensus was reached.

### Statistical analysis

The primary outcome of the meta-analysis was the predictive value of blood cell counts for irAEs. In instances where studies reported median and range for continuous blood cell counts instead of mean and standard deviation (SD), the authors utilized the formula provided by Hozo et al. to convert these values into means and SDs (16). The SMD was employed to assess the differences in continuous blood cell counts between the irAE and non-irAE groups. For studies that provided categorized blood cell counts based on specified cutoff values, the authors calculated the OR and 95% CI. The authors summarized crude and adjusted ORs to report the pooled ORs and corresponding 95% CIs. To further investigate the sources of heterogeneity between studies, subgroup analyses were conducted, considering the following potential confounding factors: cutoff values, irAEs type, cancer type, ICI type, and patient ethnicity. Statistical heterogeneity was assessed using the I² statistic, where I²≥50% indicated the presence of heterogeneity. A fixed-effects model was applied in the absence of heterogeneity; otherwise, a random-effects model was utilized. Sensitivity analyses were conducted by systematically omitting individual studies to evaluate their impact on the overall results. Publication bias was assessed using the Egger test. Statistical analyses were performed using Stata15.0 software.

## Results

### Study selection

**Figure 1:**
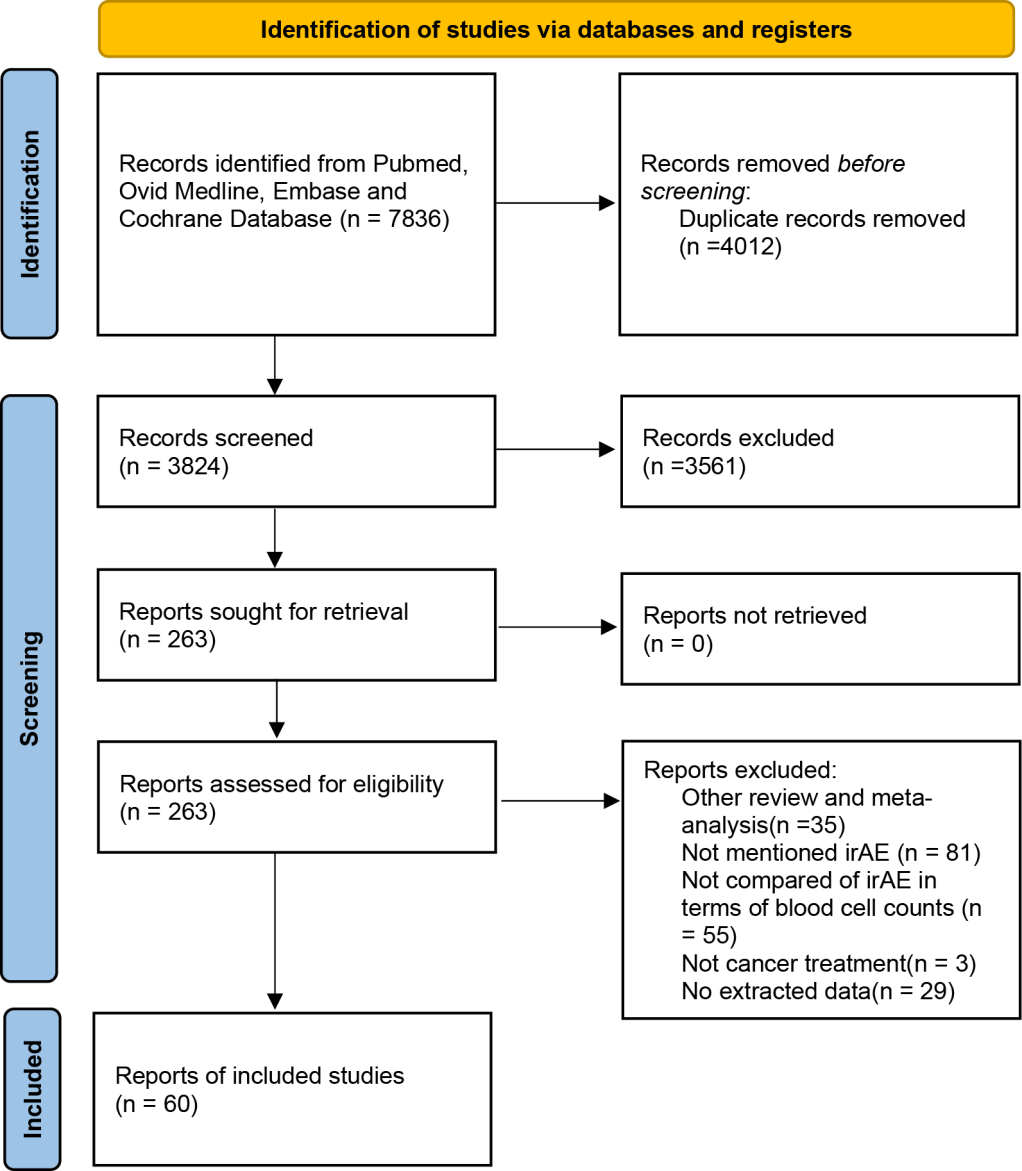
The flowchart of study screening.

A systematic search conducted across four databases identified 7836 potential studies. After removing duplicates and a comprehensive review of titles, abstracts, and full texts, 60 studies (17–76) were ultimately included in the analysis (**Figure 1**).

### Research characteristics

**Table 1** presents the features of the 60 studies included in this analysis, all published from 2018 to 2024, with 42 originating from Asia. Among these, 23 studies were from China, 17 from Japan, and 1 each from South Korea and Singapore. The remaining 18 studies were conducted in the United States (10 studies), Spain(2 study), Australia (2 study), Belgium (1 study), Germany (1 study), Switzerland (1 study) and Italy (1 study). Regarding cancer types, 22 studies specifically recruited patients with lung cancer, while 5 studies focused exclusively on liver cancer. Additionally, 4 studies included only renal cell carcinoma or urothelial carcinoma, 2 studies exclusively involved melanoma, and 1 study each targeted esophageal cancer, pancreatic cancer, gastric cancer, and head and neck squamous cell carcinoma. The remaining 24 studies included mixed cancer populations. Among the 60 studies, 21 focused exclusively on the irAEs associated with PD-1 inhibitors, while 6 assessed the irAEs linked to PD-L1 inhibitors. Additionally, 15 studies examined the irAEs related to both PD-1 and PD-L1 inhibitors. The remaining 18 studies included assessments of irAEs from PD-1/PD-L1 and CTLA-4 inhibitors. In terms of the types of irAEs, 43 studies assessed all categories of irAEs, while 6 studies specifically reported on cardiovascular adverse events, 4 focused solely on immune-related pneumonia, 3 exclusively on dermatologic adverse events, 2 on endocrine adverse events, and 1 each on colitis-related and renal adverse events.

**Table 1:** Characteristics of the included studies.

Author	Published year	Country	Cancer	Immune checkpoint inhibitors	irAE type	Peripheral blood biomarker
Dwight H Owen	2018	United States	lung cancer	Nivolumab, Pembrolizumab,Atezolizumab	All types of irAE	NLR, PLR
Yoshiyuki Nakamura	2019	Japan	melanoma	Nivolumab, Pembrolizumab	All types of irAE	ANC, ALC, AMC, AEC, NLR
Alberto Pavan	2019	Italy	lung cancer	Nivolumab, Pembrolizumab,Atezolizumab	All types of irAE	NLR, PLR
Yu Nakanishi	2019	Japan	lung cancer	Nivolumab, Pembrolizumab	Interstitial lung disease	ANC, ALC, NLR
Jun Fukihara	2019	Japan	lung cancer	Nivolumab, Pembrolizumab	Pneumonitis	NLR
Yeonghee Eun	2019	Korea	lung cancer, melanoma, lymphoma and others	Pembrolizumab	All types of irAE	ANC, NLR
Koichiro Ogihara	2020	Japan	urothelial carcinoma	Pembrolizumab	ir-SAE	NLR
Lihong Peng	2020	China	lung cancer	Nivolumab, Pembrolizumab,Toripalimab, Sintilimab	All types of irAE	NLR
Shilpa Grover	2020	United States	melanoma	Nivolumab, Pembrolizumab,Ipilimumab	Colitis	NLR
Kazuo Kobayashi	2020	Japan	renal cell carcinoma	Nivolumab	All types of irAE	ANC, ALC, PLT, NLR, PLR
Ganessan Kichenadasse	2020	Australia	lung cancer	Atezolizumab	All types of irAE	NLR
Xiangling Chu	2020	China	lung cancer	Not specified, including anti–PD-1, anti-PD-L1 inhibitors	Pneumonitis	ANC, ALC, AMC, AEC
Zsofia D Drobni	2020	United States	lung cancer, melanoma, renal cell carcinoma, head and neck carcinoma and others	Not specified, including anti–PD-1, anti-PD-L1, anti-CTLA4 inhibitors	Myocarditis	ANC, ALC, AMC, PLT, NLR
Melissa Y Y Moey	2020	United States	lung cancer	Nivolumab, Pembrolizumab,Atezolizumab	Major adverse cardiac events	PLT, NLR
Ryosuke Matsukane	2021	Japan	lung cancer, renal cell carcinoma, head and neck carcinoma, melanoma	Nivolumab, Pembrolizumab	All types of irAE	NLR, PLR
Eduard Roussel	2021	Belgium	renal cell carcinoma	Nivolumab	All types of irAE	NLR
Xiaona Fan	2021	China	gastric and colorectal cancers	Not specified, including anti–PD-1 inhibitor	All types of irAE	NLR, PLR, MLR
Pei Yi Lee	2021	Singapore	lung cancer, renal cell carcinoma, nasopharyngeal carcinoma, melanoma	Nivolumab, Pembrolizumab,Atezolizumab, Avelumab, Durvalumab, Tremelimumab	All types of irAE	ANC, ALC, PLT, NLR, PLR
Despina Michailidou	2021	United States	lung, skin, genitourinary, gastrointestinal, sarcoma, hematological malignancy, head and neck, breast carcer	Nivolumab, Pembrolizumab,Cemiplimab, Atezolizumab, Durvalumab, Avelumab, Ipilimumab, Tremelimumab	All types of irAE	ANC, ALC, AMC, NLR, PLR, MLR
Ashish Manne	2021	United States	lung cancer, melanoma	Nivolumab, Pembrolizumab,Atezolizumab, Durvalumab, Ipilimumab,	All types of irAE	ANC, ALC, PLT, NLR, PLR
Airi Fujimoto	2021	Japan	lung cancer	Nivolumab, Pembrolizumab,Atezolizumab	All types of irAE	ANC, ALC, NLR
Rilan Bai	2021	China	lung cancer, melanoma, liver cancer, esophageal cancer, urothelial cancer, gastric cancer, hypopharyngeal cancer, nasopharyngeal cancer, colon cancer, pancreatic cancer, orbital malignancy	Nivolumab, Pembrolizumab,Toripalimab, Sintilimab, Tislelizumab, Camrelizumab,Atezolizumab, Ipilimumab	All types of irAE	AEC, PLT
Lea Daniello	2021	Germany	lung cancer	Nivolumab, Pembrolizumab,Atezolizumab, Durvalumab	All types of irAE	NLR
Dan-Yun Ruan	2021	China	advanced gastric cancer	Toripalimab	All types of irAE	NLR
Yuequan Shi	2021	China	lung cancer	Not specified, including anti–PD-1, anti-PD-L1, anti-CTLA4 inhibitors	All types of irAE	ANC, ALC, AEC, NLR, PLR
Shinobu Takayasu	2022	Japan	lung cancer, renal-urinary cancer, head and neck cancer, malignant melanoma, gastric cancer, esophageal cancer and others	Nivolumab, Pembrolizumab,Atezolizumab, Avelumab, Durvalumab, Ipilimumab	Adrenal insufficiency	AEC
Kei Sonehara	2022	Japan	lung cancer	Nivolumab, Pembrolizumab,Atezolizumab	All types of irAE	NLR, PLR
Toshifumi Tada	2022	Japan	hepatocellular carcinoma	Atezolizumab	All types of irAE	NLR
Mioko Matsuo	2022	Japan	head and neck squamous cell carcinoma	Nivolumab	All types of irAE	NLR, PLR
Lijun Zhao	2022	China	lung, esophagus, gastrointestinal	Nivolumab, Pembrolizumab,Camrelizumab, Toripalimab	ir-SAE	NLR, PLR
Manuel Sánchez Cánovas	2022	Spain	melanoma and lung cancer	Nivolumab, Pembrolizumab,Atezolizumab, Durvalumab	Thrombosis	NLR
Xue Chen	2022	China	solid tumors	Not specified, including anti–PD-1, anti-PD-L1 inhibitor	Cardiotoxicity	NLR, PLR, MLR
Xiaohui Jia	2022	China	lung cancer	Not specified	Pneumonitis	ANC, ALC, AEC, PLT, NLR, PLR
Yingying Yu	2022	China	liver Cancer	Nivolumab, Camrelizumab, Sintilimab	All types of irAE	ANC, ALC, AMC, PLT, NLR, PLR
Afaf Abed	2022	Australia	lung cancer	Nivolumab, Pembrolizumab,Atezolizumab	All types of irAE	ALC, NLR, PLR
Hiroyuki Inoue	2022	Japan	esophageal cancer	Nivolumab	All types of irAE	ALC, NLR, PLR, MLR
Xiaojuan Lu	2022	China	lung cancer	Not specified, including anti–PD-1 inhibitor	All types of irAE	NLR, PLR
Yan Ma	2022	China	lung, esophageal carcinoma, liver cancer, head and neck cancer, genital system cancer, colorectal cancer, gastric carcinoma, urogenital carcinoma, cutaneous soft tissue carcinoma, melanoma, gallbladder carcinoma and bile duct carcinoma	Nivolumab, Sintilimab, Camrelizumab, Atezolizumab	All types of irAE	AEC, NLR, PLR
Zhening Zhang	2022	China	esophageal, gastric, colon cancer	Nivolumab, Pembrolizumab,Zimberelimab, Camrelizumab, Sintilimab, Tislelizumab, Toripalimab, Atezolizumab, Sugemalimab, Envafolimab, Nivolumab, Ipilimumab, Cadolinimab	All types of irAE	NLR, PLR, LMR
Si Wu	2022	China	lung cancer, stomach cancer, esophageal cancer, liver cancer, colorectal and others	Nivolumab, Pembrolizumab,Camrelizumab, Sintilimab, Toripalimab, Tislelizumab, Atezolizumab, Durvalumab	Cardiovascular adverse events	NLR
Ako Gannichida	2022	Japan	lung cancer, renal cell carcinoma, head and neck carcinoma, melanoma, gastric cancer	Nivolumab	Hypothyroidism	NLR
Cho-Han Chiang	2022	United States	head and neck cancer, gastrointestinal cancer, hepatobiliary cancer, pancreatic cancer, lung cancer, skin cancer, breast cancer, gynecologic cancer, renal and genitourinary, bone and connective tissue and others	Not specified, including anti–PD-1, anti-PD-L1, anti-CTLA4 inhibitors	Cardiotoxicity	PLR
Zhiyao Bao	2022	China	lung cancer	Nivolumab, Pembrolizumab, Toripalimab, Cindilimab, Atezolizumab, Durvalumab Tislelizumab, Camrelizumab	Renal	ANC, ALC, AMC, AEC, NLR, PLR, LMR
Yan Wu	2022	China	lung cancer	Not specified, including anti–PD-1, anti-PD-L1 inhibitor	All types of irAE	AEC, NLR
Yue Linda Wu	2022	United States	hepatocellular carcinoma	Atezolizumab	All types of irAE	NLR, PLR
Cassie Pan	2023	United States	head and neck squamous cell carcinoma, salivary gland cancer	Pembrolizumab	ir-SAE	ANC, ALC, NLR
Xin Qiu	2023	China	pancreatic cancer	Pembrolizumab, Sintilimab, Toripalimab	All types of irAE	NLR, PLR, LMR
Airi Fujimoto	2023	Japan	lung cancer	Nivolumab, Pembrolizumab, Atezolizumab, Ipilimumab	All types of irAE	NLR, PLR
Masafumi Haraguchi	2023	Japan	lung cancer, urological cancer, melanoma, head and neck cancer, gastric cancer	Nivolumab, Pembrolizumab,Atezolizumab, Ipilimumab	All types of irAE	ALC, AEC, NLR
Wei-Ting Hu	2023	China	lung cancer	Not specified, including anti–PD-1 inhibitor	All types of irAE	ANC, ALC, AEC, NLR
Tarun Mehra	2023	Switzerland	lung cancer, melanoma, renal cell carcinoma, head and neck carcinoma, hepatocellular carcinoma, urothelial carcinoma, hodgkin‐lymphoma, colorectal cancer	Nivolumab, Pembrolizumab,Atezolizumab, Ipilimumab	All types of irAE	AEC
Jiayi Gao	2023	China	lung cancer	Not specified, including anti–PD-1, anti-PD-L1 inhibitor	All types of irAE	NLR, PLR, LMR
Weitong Gao	2023	China	lung cancer	Nivolumab, Pembrolizumab,Camrelizumab, Sintilimab, Tislelizumab, Toripalimab, Atezolizumab, Durvalumab, Ipilimumab	All types of irAE	ANC, ALC, AMC, AEC, NLR, PLR, MLR
Sirish Dharmapuri	2023	United States	hepatocellular carcinoma	Not specified, including anti–PD-1, anti-CTLA4 inhibitors	All types of irAE	NLR, PLR
Lucía Teijeira	2023	Spain	lung cancer, melanoma, renal cell carcinoma, head and neck carcinoma,urothelial carcinoma, gastric adenocarcinoma, colorectal adenocarcinoma, malignant pleural mesothelioma, pancreatic adenocarcinoma, merkel cell carcinoma	Nivolumab, Pembrolizumab,Cemiplimab, Atezolizumab, Durvalumab, Avelumab	All types of irAE	ALC
Akifumi Kuwano	2024	Japan	hepatocellular carcinoma	Atezolizumab	All types of irAE	ANC, ALC, AEC, PLT, NLR, PLR
Jingting Wang	2024	China	lung cancer, head and neck cancer, gastric carcinoma, urothelial carcinoma,colorectal cancer, reproductive systemcancer, liver cancer, gallbladder carcinoma and bile duct carcinoma, melanoma and others	Nivolumab, Pembrolizumab,Camrelizumab, Sintilimab, Atezolizumab, Durvalumab	All types of irAE	AEC, NLR, MLR
Meng Yang	2024	China	urothelial carcinoma	Tislelizumab	All types of irAE	NLR, PLR, MLR
Baishen Zhang	2024	China	lung cancer	Atezolizumab, Durvalumab	All types of irAE	NLR, PLR
Masahiko Sue	2024	Japan	lung cancer, gastrointestinal cancer, head and neck cancer, kidney cancer,melanoma, liver cancer, genital cancerand others	Nivolumab, Pembrolizumab,Atezolizumab, Durvalumab, Avelumab, Ipilimumab	All types of irAE	NLR

irAE, immune-related adverse event; ANC, absolute neutrophil count; ALC, absolute lymphocyte count; AMC, absolute monocyte count; AEC, absolute eosinophil count; NLR, neutrophil to lymphocyte ratio; PLR, platelet to lymphocyte ratio; MLR, monocyte to lymphocyte ratio; LMR, lymphocyte to monocyte ratio.

### Meta-analysis

Our meta-analysis comprised 16,736 patients, featuring a median sample size of 169 per study, ranging from 41 to 1,548 (**Supplementary Table 2**). A total of 4210 irAE cases were documented, resulting in a median incidence rate of 31.71%, with rates ranging from 3.62% to 74.63%. 35 studies reported the incidence rates of various subtypes of irAEs (**Supplementary Table 3**). Dermatologic disorders were the most commonly observed irAE, with incidence rates varying between 2.57% and 58.54%. The incidence rates for pneumonia, endocrine disorders, gastrointestinal conditions, and liver injury were 0.69% to 24.83%, 1.79% to 31.11%, 0.97% to 20.30%, and 0.26% to 25.21%, respectively.

### Predictive significance of continuous blood cell counts for irAEs

**Figure 2:**
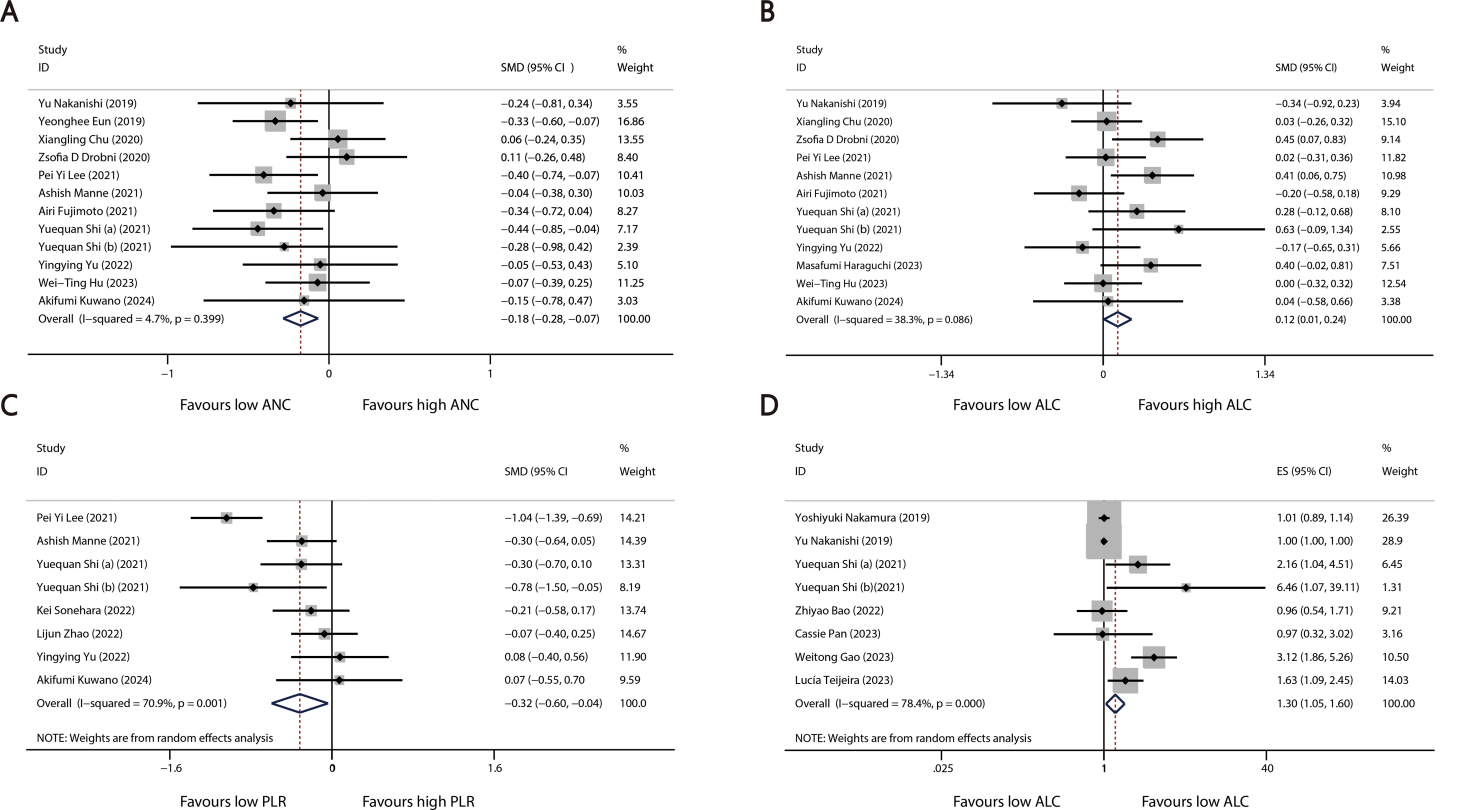
Forest plots comparing the association between continuous blood cell counts and irAEs: **(A)** Comparison of mean continuous ANC between groups. **(B)** Comparison of mean continuous ALC between groups. **(C)** Comparison of mean continuous PLR between groups. **(D)** Pooled ORs derived from continuous ALC data.

A total of 28 studies reported continuous blood cell counts for both the irAE and non-irAE groups, with 21 studies providing baseline blood cell count levels for each group and 12 studies presenting ORs for continuous blood cell counts in predicting irAEs. By synthesizing all studies reporting continuous blood cell counts to predict irAEs, we found that higher ALC (SMD=0.12, 95%CI=0.01-0.24, **Table 2**, **Figure 2B**) (OR=1.30, 95%CI=1.05-1.60, **Table 3**, **Figure 2D**), along with lower ANC (SMD=-0.18 95%CI=-0.28 to -0.07, **Table 2**, **Figure 2A**) and PLR (SMD=-0.32 95%CI=-0.60 to -0.04, **Table 2**, **Figure 2C**), were associated with a higher incidence of irAEs.

**Table 2:** Meta-analysis investigating the association between continuous blood cell counts and irAEs： Comparison of mean values for continuous variables between groups.

Blood cell	Studies (n)	SMD (95% CI)	p-value	I^2^	p-value Egger’s test
**ANC**	11	-0.18 (-0.28, -0.07)	0.001	4.70	0.901
**ALC**	11	0.12 (0.01,0.24)	0.037	38.30	0.887
AMC	3	0.16 (-0.05,0.37)	0.125	8.60	0.623
AEC	5	0.13 (-0.20,0.46)	0.441	72.20	0.427
PLT	6	-0.11 (-0.28,0.05)	0.174	0.00	0.583
NLR	17	-0.38 (-1.04,0.27)	0.249	98.50	0.812
**PLR**	7	-0.32 (-0.60,-0.04)	0.026	70.90	0.827

Bolded values indicate p < 0.05.

**Table 3:** Meta-analysis investigating the association between continuous blood cell counts and irAEs： Comparison of ORs for continuous variable values between groups.

Blood cell	Studies (n)	OR (95% CI)	p-value	I ^2^ (%)	p-value Egger’s test
ANC	5	0.95 (0.87,1.04)	0.249	66.5	0.338
**ALC**	7	1.30 (1.05,1.60)	0.016	78.4	0.049
AMC	3	0.54 (0.21,1.38)	0.198	72.8	0.175
AEC	6	1.49 (0.66,3.32)	0.335	58.5	0.455
NLR	10	1.02 (0.97,1.07)	0.511	55.5	0.153
PLR	3	0.998 (0.995, 1.002)	0.356	61.0	0.225
MLR	3	0.90 (0.81, 1.01)	0.069	0.0	0.500

Bolded values indicate p < 0.05.

### Predictive significance of categorized blood cell counts for irAEs

**Figure 3:**
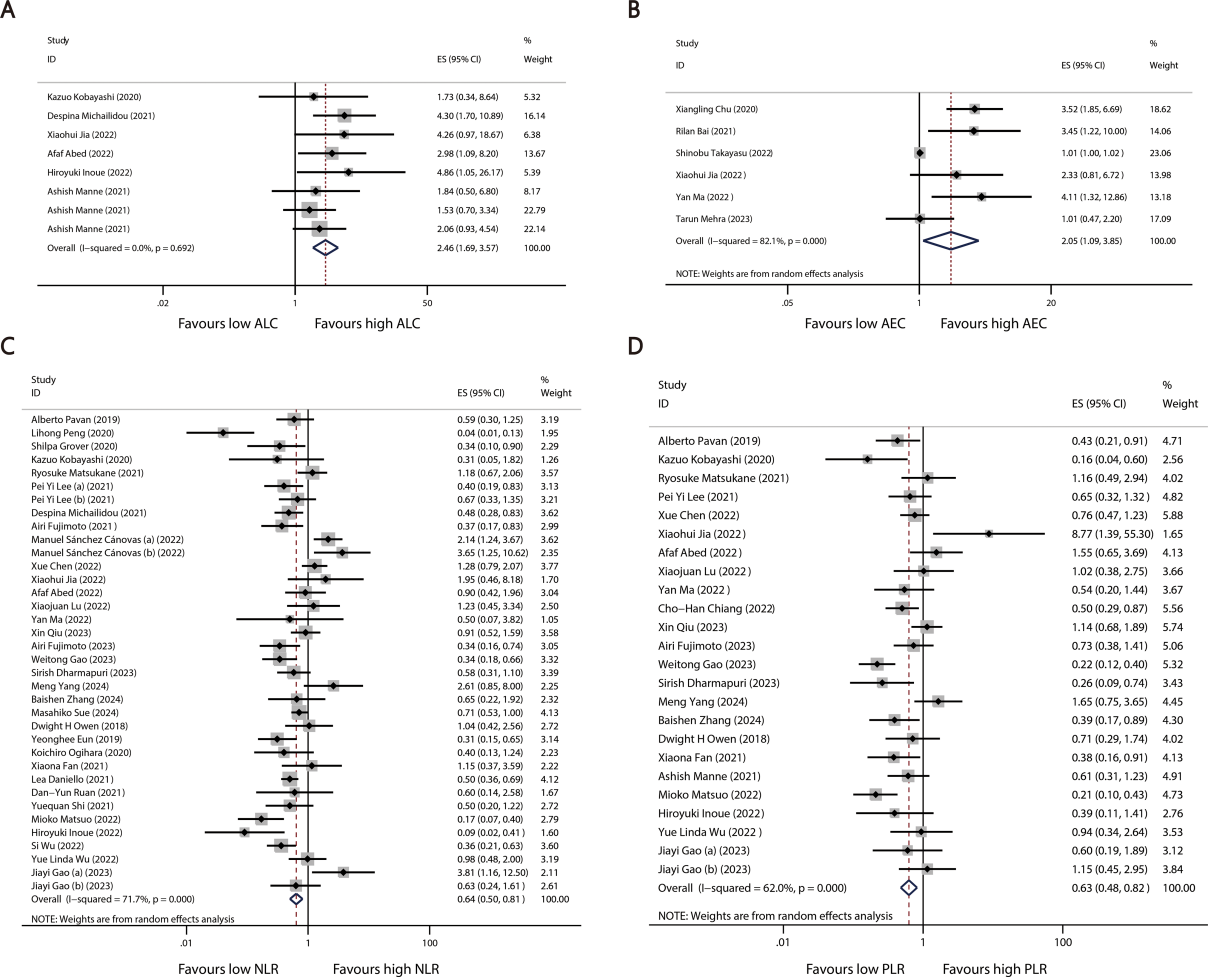
Forest plots comparing the association between categorized blood cell counts and irAEs: **(A)** ALC **(B)** AEC **(C)** NLR **(D)** PLR.

A total of 40 studies reported categorized blood cell counts for both the irAE and non-irAE groups, with 20 of these studies providing data on the number of patients with lower or higher blood cell counts in each group and 28 studies reporting calculated ORs for categorized blood cell counts in predicting irAEs using either univariate or multivariate models. By synthesizing all studies that reported categorized blood cell counts in predicting irAEs, we found that higher ALC (OR=2.46 95%CI=1.69-3.57, **Table 4**, **Figure 3A**) and AEC (OR=2.05 95%CI=1.09-3.85, **Table 4**, **Figure 3B**), as well as lower NLR (OR=0.64 95%CI=0.50-0.81 **Table 4**, **Figure 3C**) and PLR (OR=0.63 95%CI=0.48-0.82, **Table 4**, **Figure 3D**), were associated with a higher incidence of irAEs.

**Table 4:** Meta-analysis investigating the association between categorized blood cell counts and irAEs.

Blood cell	Studies (n)	OR (95% CI)	p-value	I ^2^ (%)	p-value Egger’s test
ANC	3	0.68 (0.41,1.13)	0.141	0.0	0.903
**ALC**	6	2.46 (1.69,3.57)	0.000	0.0	0.348
**AEC**	6	2.05 (1.09,3.85)	0.026	82.1	0.032
PLT	4	0.85 (0.45,1.61)	0.623	72.7	0.845
**NLR**	33	0.64 (0.50,0.81)	0.000	71.7	0.686
**PLR**	23	0.63 (0.48,0.82)	0.001	62.0	0.776
MLR	8	0.82 (0.49,1.38)	0.461	77.4	0.669

Bolded values indicate p < 0.05.

### Subgroup analysis

To explore potential sources of heterogeneity among the studies, we performed a subgroup analysis based on categorized blood cell counts. Among the different cutoff values for blood cell counts, an ALC of 2 or higher was significantly associated with an increased incidence of irAEs (OR = 2.28, 95% CI = 1.27–4.07, **Table 5**). No optimal cutoff value has yet been identified for AEC. An NLR of 5 or 3 or lower was significantly associated with an increased incidence of irAEs (OR = 0.39, 95% CI = 0.29–0.53, **Table 6**; OR = 0.63, 95% CI = 0.43–0.93, **Table 6**). Additionally, a PLR of 180 or lower was significantly associated with an increased incidence of irAEs (OR = 0.65, 95% CI = 0.45–0.95, **Table 7**).

**Table 5:** Subgroup Analysis of Categorized ALC.

Subgroup	N studies	OR (95% CI)	I^2^ (%)	p-value for heterogeneity
cutoff				
0.6	1	1.84 (0.50, 6.79)	–	0.360
1	1	1.53 (0.70, 3.34)	–	0.286
1.015	1	4.86 (0.97, 24.21)	–	0.054
1.635	1	4.26 (0.97, 18.67)	–	0.054
**2**	**3**	**2.28 (1.27, 4.07)**	**0.0**	**0.006**
2.6	1	4.30 (1.70, 10.88)	–	0.002
irAE type				
**All types of irAE**	**5**	**2.36 (1.61, 3.48)**	**0.0**	**0.000**
pneumonitis	1	4.26 (0.97, 18.67)	–	0.054
cancer type				
renal Cell Carcinoma	1	1.73 (0.34, 8.64)	–	0.507
**mixed cancer**	**2**	**2.19 (1.40, 3.43)**	**0.0**	**0.001**
**lung cancer**	**2**	**3.34 (1.45, 7.68)**	**0.0**	**0.005**
esophageal cancer	1	4.86 (0.97, 24.21)	–	0.054
drug				
PD-1	2	2.91 (0.93, 9.10)	0.0	0.066
PD-1/PD-L1	1	2.98 (1.09, 8.17)	–	0.034
**PD-1/PD-L1/CTLA-4**	**3**	**2.32 (1.51, 3.56)**	**0.0**	**0.000**
region				
**Asian**	**3**	**3.36 (1.36, 8.27)**	**0.0**	**0.009**
**non-Asian**	**3**	**2.30 (1.53, 3.47)**	**0.0**	**0.000**

Bolded values indicate p < 0.05.

**Table 6:** Subgroup Analysis of Categorized NLR.

Subgroup	N studies	OR (95% CI)	I^2^ (%)	p-value for heterogeneity
cutoff				
2	1	0.91 (0.52, 1.59)	–	0.741
2.7	1	0.60 (0.14, 2.58)	–	0.492
2.86	1	0.37 (0.17, 0.82)	–	0.014
**3**	**5**	**0.39 (0.29, 0.53)**	**0.0**	**0.000**
3.01	1	3.65 (1.25, 10.64)	–	0.018
3.1	1	1.28 (0.79, 2.07)	–	0.315
3.2	2	0.87 (0.24, 3.19)	83.5	0.830
3.28	1	1.95 (0.46, 8.18)	–	0.363
3.35	1	0.40 (0.13, 1.24)	–	0.111
3.4	1	0.31 (0.05, 1.87)	–	0.202
3.401	1	0.09 (0.02, 0.41)	–	0.002
3.56	1	1.23 (0.45, 3.34)	–	0.687
3.8	1	1.18 (0.67, 2.07)	–	0.564
4	2	1.21 (0.41, 3.55)	91.6	0.735
**5**	**12**	**0.63 (0.43, 0.93)**	**65.4**	**0.021**
5.3	1	0.48 (0.28, 0.83)	–	0.008
6.505	1	0.17 (0.07, 0.41)	–	0.000
8.58	1	0.501 (0.07, 3.81)	–	0.504
irAE type				
**All types of irAE**	**27**	**0.58 (0.46, 0.73)**	**63.5**	**0.000**
colitis	1	0.34 (0.11, 1.02)	–	0.054
thrombosis	1	2.39 (1.47, 3.87)	–	0.000
cardiovascular injury	2	0.68 (0.20, 2.37)	91.4	0.549
pneumonitis	1	1.95 (0.46, 8.18)	–	0.363
ir-SAE	1	0.40 (0.13, 1.24)	–	0.111
cancer type				
**lung cancer**	**13**	**0.59 (0.40, 0.86)**	**67.7**	**0.006**
melanoma	1	0.34 (0.11, 1.02)	–	0.054
renal cell carcinoma	1	0.31 (0.05, 1.87)	–	0.202
mixed cancer	11	0.71 (0.48, 1.06)	79.9	0.097
pancreatic cancer	1	0.91 (0.52,1.59)	–	0.741
hepatocellular carcinoma	2	0.73 (0.44, 1.22)	13.9	0.236
urothelial carcinoma	2	1.02 (0.16, 6.43)	81.3	0.981
gastric cancer	1	0.60 (0.14, 2.58)	–	0.492
esophageal cancer	1	0.09 (0.02, 0.41)	–	0.002
drug				
**PD-1**	**12**	**0.47 (0.26, 0.87)**	**79.2**	**0.017**
PD-L1	2	0.87 (0.48, 1.57)	0.0	0.635
PD-1/PD-L1	9	1.00 (0.63, 1.58)	77.0	0.993
**PD-1/CTLA-4**	**2**	**0.51 (0.29, 0.88)**	**35.8**	**0.015**
**PD-1/PD-L1/CTLA-4**	**8**	**0.50 (0.38, 0.66)**	**71.7**	**0.000**
region				
**Asian**	**24**	**0.57 (0.42, 0.76)**	**71.1**	**0.000**
non-Asian	9	0.83 (0.55, 1.25)	74.7	0.363

Bolded values indicate p < 0.05.

**Table 7:** Subgroup Analysis of Categorized PLR.

Subgroup	N studies	OR (95% CI)	I^2^ (%)	p-value for heterogeneity
cutoff				
114.271	1	8.77(1.39, 55.32)	–	0.021
135	3	0.93(0.44, 1.96)	69.5	0.846
143	1	0.22(0.12, 0.40)	–	0.000
150	1	0.61(0.31, 1.26)	–	0.160
156	1	0.16(0.04, 0.62)	–	0.008
163	1	0.76(0.47, 1.23)	–	0.263
**180**	**5**	**0.65(0.45, 0.95)**	**33.9**	**0.028**
180.68	1	0.54(0.20, 1.45)	–	0.221
185	1	0.39(0.17, 0.89)	–	0.026
200	1	1.02(0.38, 2.74)	–	0.969
237	1	0.71(0.29, 1.74)	–	0.454
240	1	1.16(0.47, 2.84)	–	0.745
243	1	0.39(0.11, 1.40)	–	0.148
300	2	0.50(0.14, 1.75)	66.0	0.277
320	1	0.21(0.10, 0.44)	–	0.000
436	1	0.89(0.43, 1.83)	–	0.744
irAE type				
**All types of irAE**	**20**	**0.60(0.45, 0.80)**	**61.0**	**0.001**
**cardiovascular injury**	**2**	**0.63(0.42, 0.95)**	**20.8**	**0.027**
pneumonitis	1	8.77(1.39, 55.32)	–	0.021
cancer type				
lung cancer	9	0.72(0.44, 1.17)	68.6	0.182
renal cell carcinoma	1	0.16(0.04, 0.62)	–	0.008
**mixed cancer**	**8**	**0.55(0.39, 0.76)**	**42.6**	**0.000**
pancreatic cancer	1	1.14(0.68, 1.90)	–	0.615
hepatocellular carcinoma	2	0.50(0.14, 1.75)	66.0	0.277
urothelial carcinoma	1	1.65(0.75, 3.64)	–	0.215
esophageal cancer	1	0.39(0.11, 1.40)	–	0.148
drug				
PD-1	8	0.61(0.33, 1.11)	74.6	0.106
PD-L1	2	0.57(0.24, 1.35)	41.6	0.203
PD-1/PD-L1	6	0.74(0.55, 1.01)	4.4	0.055
PD-1/CTLA-4	1	0.26(0.09, 0.75)	–	0.012
PD-1/PD-L1/CTLA-4	6	0.61(0.34, 1.07)	73.0	0.083
region				
**Asian**	**16**	**0.63(0.44, 0.91)**	**68.9**	**0.012**
**non-Asian**	**7**	**0.61(0.42, 0.88)**	**34.1**	**0.009**

Bolded values indicate p < 0.05.

Additionally, we performed a subgroup analysis based on specific types of irAEs. The results indicated that higher AEC values were associated with an increased incidence of pneumonitis (OR = 3.15, 95% CI = 1.82–5.45, **Table 8**), while lower PLR values were associated with an increased incidence of cardiovascular injury (OR = 0.65, 95% CI = 0.45–0.95, **Table 7**).

**Table 8:** Subgroup Analysis of Categorized AEC.

Subgroup	N studies	OR (95% CI)	I^2^ (%)	p-value for heterogeneity
cutoff				
0.045	1	4.11(1.32, 12.86)	–	0.015
0.125	1	3.52(1.85, 6.69)	–	0.000
0.155	1	2.33(0.81, 6.72)	–	0.118
0.175	1	3.45(1.22, 10.00)	–	0.021
0.198	1	1.01(1.00, 1.02)	–	0.049
0.2	1	1.01(0.47, 2.20)	–	0.974
irAE type				
**pneumonitis**	**2**	**3.15(1.82, 5.45)**	**0.0**	**0.000**
All types of irAE	3	2.26(0.88, 5.80)	63.7	0.089
adrenal insufficiency	1	1.01(1.00,1.02)	–	0.049
cancer type				
**lung cancer**	**2**	**3.15(1.82, 5.45)**	**0.0**	**0.000**
mixed cancer	4	1.66(0.85, 3.25)	72.9	0.142
drug				
**PD-1/PD-L1**	**2**	**3.65(2.09, 6.39)**	**0.0**	**0.000**
PD-1/PD-L1/CTLA-4	4	1.42(0.83, 2.45)	60.7	0.204
region				
**Asian**	**5**	**2.42(1.10, 5.33)**	**85.7**	**0.028**
non-Asian	1	1.01(0.47, 2.20)	–	0.974

Bolded values indicate p < 0.05.

Subgroup analysis based on cancer type revealed that higher ALC (OR = 3.34, 95% CI = 1.45–7.68, **Table 5**) and AEC (OR = 3.15, 95% CI = 1.82–5.45, **Table 8**) were associated with an increased incidence of irAEs in lung cancer patients. Additionally, lower NLR was associated with an increased incidence of irAEs in lung cancer patients (OR = 0.59, 95% CI = 0.40–0.86, **Table 6**).

We conducted a subgroup analysis based on the type of ICI. Among the 40 studies that evaluated continuous blood cell counts, 12 studies evaluated PD-1 inhibitors alone, 3 studies evaluated PD-L1 inhibitors alone, 10 studies assessed both PD-1 and PD-L1 inhibitors, 2 studies evaluated both PD-1 and CTLA-4 inhibitors, and 13 studies evaluated PD-1, PD-L1, and CTLA-4 inhibitors simultaneously. In patients receiving PD-1 inhibitors, lower NLR values were associated with a higher incidence of irAEs (OR=0.47, 95% CI=0.26-0.87, **Table 6**).

Finally, the subgroup analysis based on the publication region categorized the studies into those from Asian and non-Asian countries. In Asian countries, higher ALC values (OR=3.36, 95% CI=1.36-8.27, **Table 5**) and AEC values (OR=2.42, 95% CI=1.10-5.33, **Table 8**), as well as lower NLR values (OR=0.57, 95% CI=0.42-0.76, **Table 6**) and PLR values (OR=0.63, 95% CI=0.44-0.91, **Table 7**), were associated with a higher incidence of irAEs. In non-Asian countries, higher ALC values (OR=2.30, 95% CI=1.53-3.47, **Table 5**) and lower PLR values (OR=0.61, 95% CI=0.42-0.88, **Table 7**) were also associated with an increased incidence of irAEs.

### Sensitivity analysis

We conducted a sensitivity analysis to explore potential sources of heterogeneity. For continuous ALC in predicting irAEs, heterogeneity was significantly influenced by each study, likely due to the limited number of studies included (**Supplementary Figure 1D**). All other combined results were relatively robust (**Supplementary Figure 1**).

### Quality assessment and publication bias assessment

We considered 51 studies to be of high quality, while the remaining 9 studies had NOS scores ranging from 5 to 6. Egger’s test indicated a potential publication bias for the studies calculating the combined OR for categorized AEC (P = 0.032).

## Discussion

Through an extensive meta-analysis, we analyzed 60 studies involving 16,736 cancer patients and found that peripheral blood cell counts could serve as potential biomarkers for predicting adverse events.

Lymphocytes, particularly T and B cells, play a crucial role in the pathogenesis of irAEs. T cells, especially the CD4+ and CD8+ subsets, are central to the immune responses triggered by ICIs (77–79). Immune checkpoint blockade enhances T cell activity and proliferation, potentially leading to a breakdown in self-tolerance. This overactivation may expand autoreactive T cell clones that can recognize and attack healthy tissues (80, 81). Studies have shown that some tumors and normal tissues share identical T cell receptor (TCR) sequences (82). ICIs can induce diversification of the TCR repertoire, generating autoreactive T cells that target normal tissue antigens (79). T cell infiltration into damaged tissues has also been observed in cases of myocarditis and skin toxicities (79, 83). Regulatory T cells (Tregs) play a critical role in maintaining peripheral tolerance by suppressing autoreactive T cells (84, 85). ICIs may reduce Treg populations, disrupting the balance between effector T cells and Tregs, thereby enhancing immune responses against self-antigens and promoting autoimmune phenomena (86). After ICI treatment, activated B cells may generate autoantibodies targeting self-antigens, leading to tissue damage and inflammatory responses (87). This mechanism is particularly evident in irAEs such as thyroid dysfunction, where affected patients often exhibit elevated levels of anti-thyroid autoantibodies (88). Furthermore, interactions between autoreactive T cells and B cells facilitate the formation of immune complexes, such as anti-nuclear antibody (ANA)-antigen complexes and anti-double-stranded DNA (anti-dsDNA) antibody-antigen complexes, which activate the complement system, triggering inflammation and exacerbating tissue damage (87). Additionally, pro-inflammatory cytokines produced by T cells and B cells, such as IL-17, TNF-α, and IFN-γ, amplify systemic inflammation and contribute to organ-specific damage (89, 90). The results of our meta-analysis indicate that elevated levels of peripheral blood lymphocytes are associated with an increased incidence of irAEs (OR=2.46, 95% CI=1.69-3.57). High circulating lymphocyte levels may reflect enhanced immune surveillance and signify sustained anti-tumor activity. However, this heightened immune surveillance may also lead to the recognition of self-antigens, potentially triggering autoimmune responses.

Eosinophils have traditionally been associated with allergic reactions and parasitic infections (91); however, recent research suggests that these cells also play diverse roles in anti-tumor immunity and autoimmunity. Eosinophils can recruit tumor-specific CD8+ T cells into the tumor microenvironment by secreting chemokines such as CCL5, CXCL9, and CXCL10, and induce macrophage polarization toward the M1 phenotype (92), thereby enhancing the immune system’s ability to recognize and destroy tumor cells. In a melanoma mouse model, depletion of regulatory T cells significantly increased eosinophil infiltration, which was associated with tumor regression (93). Eosinophils can also exert direct cytotoxic effects on tumor cells by releasing substances like major basic protein (MBP), demonstrating potent tumor-killing activity in melanoma cells (92, 94). Numerous studies have shown that elevated eosinophil levels are linked to stronger anti-tumor immune responses and better prognosis in patients receiving ICI therapy (95, 96). Eosinophils contribute to these processes by secreting chemokines that promote the recruitment and activation of tumor-specific CD8+ T cells (93). After ICI treatment, CD4+ T cells release interleukin-5 (IL-5), stimulating eosinophil production in the bone marrow, which subsequently leads to their accumulation in peripheral blood and infiltration into tumor tissues (93). While eosinophils may contribute to favorable therapeutic outcomes, their accumulation in healthy tissues is also implicated in the development of irAEs (90, 97). Eosinophils produce various pro-inflammatory cytokines and chemokines, including interleukin-4 (IL-4), IL-5, interleukin-13 (IL-13), and tumor necrosis factor-alpha (TNF-α) (98). These mediators not only recruit additional immune cells to sites of inflammation but also sustain and amplify the inflammatory response. In patients undergoing ICI therapy, increased eosinophil infiltration has been observed in tissues affected by irAEs, such as the skin, lungs, and gastrointestinal tract (99–101). Eosinophils express a variety of surface markers and receptors, such as CD30 and PD-L1, which facilitate their interaction with T cells and enhance a Th2-skewed immune response characterized by increased production of IL-4 and IL-5. This interaction may result in allergic-like reactions in patients receiving ICIs (102). The Th2-biased immune response can further exacerbate tissue inflammation and damage, contributing to the development of irAEs. Our meta-analysis indicates that elevated peripheral blood eosinophil levels are associated with an increased incidence of irAEs(OR=2.05, 95%CI=1.09-3.85). Initially, high eosinophil levels may correlate with effective anti-tumor immune responses. However, their prolonged presence and activation can provoke harmful autoimmune reactions. ICI-related pneumonitis (CIP) often presents as bilateral ground-glass opacities or nodules, with eosinophils frequently observed (100). Additionally, a small number of eosinophils can be detected in bronchoalveolar lavage fluid (103). Our subgroup analysis revealed that elevated AEC are associated with an increased incidence of pneumonia (OR = 3.15, 95% CI = 1.82-5.45), suggesting that eosinophils may play a significant role in the development of CIP. Although the specific mechanisms underlying the relationship between eosinophils and CIP remain unclear, it is essential to be vigilant regarding the potential risk of CIP in patients with baseline eosinophilia.

NLR and PLR are recognized markers of inflammation, playing a pivotal role in assessing the effectiveness and prognosis of immune checkpoint inhibitor therapy (8, 104). In our study, a low NLR(OR=0.64, 95%CI=0.50-0.81)and PLR(OR=0.63, 95%CI=0.48-0.82)were associated with an increased incidence of irAEs. An elevated NLR typically indicates a chronic inflammatory state characterized by increased levels of polymorphonuclear myeloid-derived suppressor cells (PMN-MDSCs) and tumor-associated neutrophils (62), which can suppress the anti-tumor function of lymphocytes, thereby weakening the immune response against tumors (105, 106). Besides their role in hemostasis, platelets also play a critical role in immune modulation (107). They release pro-inflammatory cytokines and chemokines upon activation, such as interleukin-1 (IL-1), TNF-α, and interleukin-6 (IL-6) (108). These mediators are essential for enhancing local inflammatory responses and recruiting immune cells, including monocytes and T cells, to sites of inflammation (109). Platelet activation is associated with chronic inflammatory states and can inhibit lymphocyte function, impairing the anti-tumor immune response (110). In contrast, a high lymphocyte count typically signifies a robust immune response capable of effectively combating tumors. Thus, lower NLR and PLR, indicating reduced neutrophil and platelet levels alongside a high lymphocyte count, reflect sustained anti-tumor activity and suggest an increased risk of developing irAEs. Although previous literature indicates that elevated PLR is associated with an increased risk of cardiovascular events (111, 112), our subgroup analysis revealed that a lower PLR (OR = 0.65, 95% CI = 0.45-0.95) is associated with a higher incidence of immune-related cardiovascular adverse events. It is important to note that immune-related cardiovascular events differ significantly from traditional cardiovascular events in their underlying mechanisms, placing greater emphasis on the role of immune and inflammatory responses in disease development. The bidirectional changes in PLR may reflect the complex immune-inflammatory mechanisms involved in different cardiovascular events, indicating that patients with varying PLR levels may face distinct immune-related risk profiles.

This study demonstrates that peripheral blood cell counts (such as ALC, AEC, NLR, and PLR) are strongly associated with irAEs and can serve as early predictive markers for patients undergoing immunotherapy. Regular monitoring of these biomarkers enables the timely identification of high-risk patients and facilitates early intervention. For instance, an increase in lymphocyte count may suggest immune system overactivation (71), potentially signaling the onset of irAEs, while elevated eosinophil count may be closely linked to immune-related pneumonia (23). These biomarkers predict the occurrence of irAEs and help assess their severity, thus guiding treatment adjustments (113). Increased NLR and PLR may indicate immune activation, enabling clinicians to adjust the immunotherapy dose or temporarily pause treatment. For patients who have already developed irAEs, regular monitoring of hematological markers helps assess immune response control and determine the need for immunosuppressive agents to manage adverse reactions. The use of hematological biomarkers supports personalized treatment management. By integrating clinical characteristics with peripheral blood cell counts, clinicians can more effectively balance treatment efficacy and safety (8, 114).

The application of these biomarkers in clinical practice faces several challenges. First, their sensitivity and specificity in predicting irAEs require further validation. Factors such as patient population, tumor type, immune checkpoint inhibitors, treatment timing, regimen, and detection methods may influence biomarker performance, resulting in variability across different populations (38, 113, 115, 116). Second, clinical thresholds for hematological biomarkers have not been standardized, and cutoff values vary significantly across studies, introducing uncertainty in their application. Our meta-analysis suggests that the optimal cutoff values for ALC, NLR, and PLR are 2×10^9^/L, 3 or 5, and 180, respectively, while no clear standard exists for AEC. These critical values are influenced by factors such as study population, leading to heterogeneity in results. Therefore, future multicenter, large-scale prospective studies are needed to establish optimal clinical standards for these biomarkers.

Our study presents several significant strengths. First, it includes a substantial sample size, comprising 60 studies and 16,736 cancer patients, thereby enhancing the reliability of our conclusions. Second, we assessed continuous and categorical blood cell counts, strengthening our findings’ robustness. Third, we performed comprehensive subgroup analyses according to varying blood cell counts cutoffs, specific irAEs (such as pneumonitis and cardiovascular injury), cancer types, ICIs, and patient demographics. Fourth, all included studies were of moderate to high quality, and sensitivity analyses further validated the stability of our results. Finally, we identified a commonly used cutoff value for blood cell counts, which offers valuable guidance for clinical practice, although this finding requires further validation.

Our study has several significant limitations that require careful consideration. First, since our analysis primarily relies on retrospective studies, there is a potential for selection bias and heterogeneity, which could affect the generalizability of our findings. Second, the substantial variability in blood cell counts cutoff values across different studies indicates a lack of consensus on an optimal threshold, thereby limiting its applicability in clinical practice. Third, this meta-analysis includes only published studies, which raises concerns about publication bias, given that unpublished negative results may skew our findings. Fourth, the scarcity of reports on specific irAE subtypes, such as cardiovascular events and pneumonia, limits our ability to evaluate the predictive capacity of blood cell counts for these adverse events. Finally, the heterogeneity among studies, especially in the analysis of continuous blood cell counts, may compromise the reliability of our results. Although subgroup and sensitivity analyses were performed to explore sources of heterogeneity, the limited number of studies and the diversity in study design, populations, and reporting methods may still leave some heterogeneity unexplained.

## Conclusion

In conclusion, our meta-analysis revealed a significant association between higher baseline ALC and AEC, and lower baseline PLR and NLR, with an increased risk of developing irAEs. However, the predictive value of blood cell counts varies across different types of irAEs, highlighting the need for additional subgroup analyses when evaluating the efficacy of peripheral biomarkers. The commonly used cutoff values (ALC = 2×10^9^/L, NLR = 3 or 5, PLR = 180) require further consensus to establish the optimal cutoff for future clinical guidance. Overall, our findings suggest that blood cell counts may serve as a valuable predictor of irAEs, and further research is warranted to evaluate its role in personalized immunotherapy management.

## Data Availability

The original contributions presented in the study are included in the article/**Supplementary Material**. Further inquiries can be directed to the corresponding author.

## References

[1] Morad G Helmink BA Sharma P Wargo JA . Hallmarks of response, resistance, and toxicity to immune checkpoint blockade. Cell. (2021) 184:5309–37. doi: 10.1016/j.cell.2021.09.020 PMC8767569 34624224

[2] Das S Johnson DB . Immune-related adverse events and anti-tumor efficacy of immune checkpoint inhibitors. J Immunother Cancer. (2019) 7:306. doi: 10.1186/s40425-019-0805-8 31730012 PMC6858629

[3] Poto R Troiani T Criscuolo G Marone G Ciardiello F Tocchetti CG . Holistic approach to immune checkpoint inhibitor-related adverse events. Front Immunol. (2022) 13:804597. doi: 10.3389/fimmu.2022.804597 35432346 PMC9005797

[4] Schneider BJ Naidoo J Santomasso BD Lacchetti C Adkins S Anadkat M . Management of immune-related adverse events in patients treated with immune checkpoint inhibitor therapy: ASCO guideline update. J Clin Oncol. (2021) 39:4073–126. doi: 10.1200/JCO.21.01440 34724392

[5] Petrelli F Grizzi G Ghidini M Ghidini A Ratti M Panni S . Immune-related adverse events and survival in solid tumors treated with immune checkpoint inhibitors: A systematic review and meta-analysis. J Immunotherapy (Hagerstown Md: 1997). (2020) 43:1–7. doi: 10.1097/CJI.0000000000000300 31574022

[6] Zhou X Yao Z Yang H Liang N Zhang X Zhang F . Are immune-related adverse events associated with the efficacy of immune checkpoint inhibitors in patients with cancer? A systematic review and meta-analysis. BMC Med. (2020) 18:87. doi: 10.1186/s12916-020-01549-2 32306958 PMC7169020

[7] Park R Lopes L Saeed A . Anti-PD-1/L1-associated immune-related adverse events as harbinger of favorable clinical outcome: systematic review and meta-analysis. Clin Trans Oncology: Off Publ Fed Spanish Oncol Societies Natl Cancer Institute Mexico. (2021) 23:100–9. doi: 10.1007/s12094-020-02397-5 32495269

[8] Chennamadhavuni A Abushahin L Jin N Presley CJ Manne A . Risk factors and biomarkers for immune-related adverse events: A practical guide to identifying high-risk patients and rechallenging immune checkpoint inhibitors. Front Immunol. (2022) 13:779691. doi: 10.3389/fimmu.2022.779691 35558065 PMC9086893

[9] Asada M Mikami T Niimura T Zamami Y Uesawa Y Chuma M . The risk factors associated with immune checkpoint inhibitor-related pneumonitis. Oncology. (2021) 99:256–9. doi: 10.1159/000512633 33477139

[10] Xu Y Fu Y Zhu B Wang J Zhang B . Predictive biomarkers of immune checkpoint inhibitors-related toxicities. Front Immunol. (2020) 11:2023. doi: 10.3389/fimmu.2020.02023 33123120 PMC7572846

[11] Jing Y Zhang Y Wang J Li K Chen X Heng J . Association between sex and immune-related adverse events during immune checkpoint inhibitor therapy. J Natl Cancer Inst. (2021) 113:1396–404. doi: 10.1093/jnci/djab035 33705549

[12] Sekine K Kanda S Goto Y Horinouchi H Fujiwara Y Yamamoto N . Change in the lymphocyte-to-monocyte ratio is an early surrogate marker of the efficacy of nivolumab monotherapy in advanced non-small-cell lung cancer. Lung Cancer (Amsterdam Netherlands). (2018) 124:179–88. doi: 10.1016/j.lungcan.2018.08.012 30268458

[13] Sung C An J Lee S Park J Lee KS Kim I-H . Integrative analysis of risk factors for immune-related adverse events of checkpoint blockade therapy in cancer. Nat Cancer. (2023) 4:844–59. doi: 10.1038/s43018-023-00572-5 37308678

[14] Lu H-R Zhu P-F Deng Y-Y Chen Z-L Yang L . Predictive value of NLR and PLR for immune-related adverse events: a systematic review and meta-analysis. Clin Trans Oncology: Off Publ Fed Spanish Oncol Societies Natl Cancer Institute Mexico. (2024) 26:1106–16. doi: 10.1007/s12094-023-03313-3 37682501

[15] Zhang W Tan Y Li Y Liu J . Neutrophil to Lymphocyte ratio as a predictor for immune-related adverse events in cancer patients treated with immune checkpoint inhibitors: a systematic review and meta-analysis. Front Immunol. (2023) 14:1234142. doi: 10.3389/fimmu.2023.1234142 37622124 PMC10445236

[16] Hozo SP Djulbegovic B Hozo I . Estimating the mean and variance from the median, range, and the size of a sample. BMC Med Res Methodol. (2005) 5:13. doi: 10.1186/1471-2288-5-13 15840177 PMC1097734

[17] Owen DH Wei L Bertino EM Edd T Villalona-Calero MA He K . Incidence, risk factors, and effect on survival of immune-related adverse events in patients with non-small-cell lung cancer. Clin Lung Cancer. (2018) 19:e893–900. doi: 10.1016/j.cllc.2018.08.008 PMC7193681 30197259

[18] Eun Y Kim IY Sun JM Lee J Cha HS Koh EM . Risk factors for immune-related adverse events associated with anti-PD-1 pembrolizumab. Sci Rep. (2019) 9:14039. doi: 10.1038/s41598-019-50574-6 31575933 PMC6773778

[19] Fukihara J Sakamoto K Koyama J Ito T Iwano S Morise M . Prognostic impact and risk factors of immune-related pneumonitis in patients with non-small-cell lung cancer who received programmed death 1 inhibitors. Clin Lung Cancer. (2019) 20:442–450 e4. doi: 10.1016/j.cllc.2019.07.006 31446020

[20] Nakamura Y Tanaka R Maruyama H Ishitsuka Y Okiyama N Watanabe R . Correlation between blood cell count and outcome of melanoma patients treated with anti-PD-1 antibodies. Jpn J Clin Oncol. (2019) 49:431–7. doi: 10.1093/jjco/hyy201 30753621

[21] Nakanishi Y Masuda T Yamaguchi K Sakamoto S Horimasu Y Nakashima T . Pre-existing interstitial lung abnormalities are risk factors for immune checkpoint inhibitor-induced interstitial lung disease in non-small cell lung cancer. Respir Investig. (2019) 57:451–9. doi: 10.1016/j.resinv.2019.05.002 31248832

[22] Pavan A Calvetti L Dal Maso A Attili I Del Bianco P Pasello G . Peripheral blood markers identify risk of immune-related toxicity in advanced non-small cell lung cancer treated with immune-checkpoint inhibitors. Oncologist. (2019) 24:1128–36. doi: 10.1634/theoncologist.2018-0563 PMC6693718 31015312

[23] Chu X Zhao J Zhou J Zhou F Jiang T Jiang S . Association of baseline peripheral-blood eosinophil count with immune checkpoint inhibitor-related pneumonitis and clinical outcomes in patients with non-small cell lung cancer receiving immune checkpoint inhibitors. Lung Cancer. (2020) 150:76–82. doi: 10.1016/j.lungcan.2020.08.015 33080551

[24] Drobni ZD Zafar A Zubiri L Zlotoff DA Alvi RM Lee C . Decreased absolute lymphocyte count and increased neutrophil/lymphocyte ratio with immune checkpoint inhibitor-associated myocarditis. J Am Heart Assoc. (2020) 9:e018306. doi: 10.1161/JAHA.120.018306 33190570 PMC7763791

[25] Grover S Dougan M Tyan K Giobbie-Hurder A Blum SM Ishizuka J . Vitamin D intake is associated with decreased risk of immune checkpoint inhibitor-induced colitis. Cancer. (2020) 126:3758–67. doi: 10.1002/cncr.32966 PMC7381363 32567084

[26] Kichenadasse G Miners JO Mangoni AA Rowland A Hopkins AM Sorich MJ . Multiorgan immune-related adverse events during treatment with atezolizumab. J Natl Compr Canc Netw. (2020) 18:1191–9. doi: 10.6004/jnccn.2020.7567 32886899

[27] Kobayashi K Iikura Y Hiraide M Yokokawa T Aoyama T Shikibu S . Association between immune-related adverse events and clinical outcome following nivolumab treatment in patients with metastatic renal cell carcinoma. In Vivo. (2020) 34:2647–52. doi: 10.21873/invivo.12083 PMC7652517 32871795

[28] Moey MYY Tomdio AN McCallen JD Vaughan LM O'brien K Naqash AR . Characterization of immune checkpoint inhibitor-related cardiotoxicity in lung cancer patients from a rural setting. JACC CardioOncol. (2020) 2:491–502. doi: 10.1016/j.jaccao.2020.07.005 34396256 PMC8352337

[29] Ogihara K Kikuchi E Shigeta K Okabe T Hattori S Yamashita R . The pretreatment neutrophil-to-lymphocyte ratio is a novel biomarker for predicting clinical responses to pembrolizumab in platinum-resistant metastatic urothelial carcinoma patients. Urol Oncol. (2020) 38:602 e1–602.e10. doi: 10.1016/j.urolonc.2020.02.005 32139290

[30] Peng L Wang Y Liu F Qiu X Zhang X Fang C . Peripheral blood markers predictive of outcome and immune-related adverse events in advanced non-small cell lung cancer treated with PD-1 inhibitors. Cancer Immunol Immunother. (2020) 69:1813–22. doi: 10.1007/s00262-020-02585-w PMC7413896 32350592

[31] Bai R Chen N Chen X Li L Song W Li W . Analysis of characteristics and predictive factors of immune checkpoint inhibitor-related adverse events. Cancer Biol Med. (2021) 18:1118–33. doi: 10.20892/j.issn.2095-3941.2021.0052 PMC8610160 34259422

[32] Daniello L Elshiaty M Bozorgmehr F Kuon J Kazdal D Schindler H . Therapeutic and prognostic implications of immune-related adverse events in advanced non-small-cell lung cancer. Front Oncol. (2021) 11:703893. doi: 10.3389/fonc.2021.703893 34268127 PMC8277237

[33] Fan X Wang D Zhang W Liu J Liu C Li Q . Inflammatory markers predict survival in patients with advanced gastric and colorectal cancers receiving anti-PD-1 therapy. Front Cell Dev Biol. (2021) 9:638312. doi: 10.3389/fcell.2021.638312 33791296 PMC8005614

[34] Fujimoto A Toyokawa G Koutake Y Kimura S Kawamata Y Fukuishi K . Association between pretreatment neutrophil-to-lymphocyte ratio and immune-related adverse events due to immune checkpoint inhibitors in patients with non-small cell lung cancer. Thorac Cancer. Aug. (2021) 12:2198–204. doi: 10.1111/1759-7714.14063 PMC8327687 34173724

[35] Lee PY Oen KQX Lim GRS Hartono JL Muthiah M Huang DQ . Neutrophil-to-lymphocyte ratio predicts development of immune-related adverse events and outcomes from immune checkpoint blockade: A case-control study. Cancers (Basel). (2021) 13(6):1308. doi: 10.3390/cancers13061308 33804050 PMC8001500

[36] Manne A Mulekar MS Escobar DE Alsayed A Sharma G Prodduturvar P . Clinical and hematological predictors of high-grade immune-related adverse events associated with immune checkpoint inhibitors. J Clin Med Res May. (2021) 13:268–75. doi: 10.14740/jocmr4511 PMC8166288 34104278

[37] Matsukane R Watanabe H Minami H Hata K Suetsugu K Tsuji T . Continuous monitoring of neutrophils to lymphocytes ratio for estimating the onset, severity, and subsequent prognosis of immune related adverse events. Sci Rep Jan 14. (2021) 11:1324. doi: 10.1038/s41598-020-79397-6 PMC7809015 33446685

[38] Michailidou D Khaki AR Morelli MP Diamantopoulos L Singh N Grivas P . Association of blood biomarkers and autoimmunity with immune related adverse events in patients with cancer treated with immune checkpoint inhibitors. Sci Rep. (2021) 11:9029. doi: 10.1038/s41598-021-88307-3 33907229 PMC8079370

[39] Roussel E Kinget L Verbiest A Debruyne PR Baldewijns M Van Poppel H . C-reactive protein and neutrophil-lymphocyte ratio are prognostic in metastatic clear-cell renal cell carcinoma patients treated with nivolumab. Urol Oncol. (2021) 39:239 e17–239 e25. doi: 10.1016/j.urolonc.2020.12.020 33485762

[40] Ruan DY Chen YX Wei XL Wang YN Wang ZX Wu HX . Elevated peripheral blood neutrophil-to-lymphocyte ratio is associated with an immunosuppressive tumour microenvironment and decreased benefit of PD-1 antibody in advanced gastric cancer. Gastroenterol Rep (Oxf). (2021) 9:560–70. doi: 10.1093/gastro/goab032 PMC8677531 34925853

[41] Shi Y Liu X Liu J Zhang D Liu X Yue Y . Correlations between peripheral blood biomarkers and clinical outcomes in advanced non-small cell lung cancer patients who received immunotherapy-based treatments. Transl Lung Cancer Res. (2021) 10:4477–93. doi: 10.21037/tlcr-21-710 PMC8743518 35070755

[42] Abed A Law N Calapre L Lo J Bhat V Bowyer S . Human leucocyte antigen genotype association with the development of immune-related adverse events in patients with non-small cell lung cancer treated with single agent immunotherapy. Eur J Cancer. (2022) 172:98–106. doi: 10.1016/j.ejca.2022.05.021 35759816

[43] Bao Z Luo L Xu T Yang J Lv M Ni L . Risk factors and prognostic role of renal adverse event in patients receiving immune checkpoint inhibitor therapy: analysis of data from a retrospective cohort study. Ann Transl Med. (2022) 10:967. doi: 10.21037/atm-22-3684 36267724 PMC9577783

[44] Canovas MS Garay DF Moran LO Perez JR Rubio CMG De Mena ML . Immune checkpoint inhibitors-associated thrombosis in patients with lung cancer and melanoma: a study of the Spanish society of medical oncology (SEOM) thrombosis and cancer group. Clin Transl Oncol. (2022) 24:2010–20. doi: 10.1007/s12094-022-02860-5 PMC9418291 35668339

[45] Chen X Jiang A Zhang R Fu X Liu N Shi C . Immune checkpoint inhibitor-associated cardiotoxicity in solid tumors: real-world incidence, risk factors, and prognostic analysis. Front Cardiovasc Med. (2022) 9:882167. doi: 10.3389/fcvm.2022.882167 35669482 PMC9163804

[46] Chiang CH Chiang CH Ma KS Hsia YP Lee YW Wu HR . The incidence and risk of cardiovascular events associated with immune checkpoint inhibitors in Asian populations. Jpn J Clin Oncol. (2022) 52:1389–98. doi: 10.1093/jjco/hyac150 PMC9721460 36208180

[47] Gannichida A Nakazawa Y Kageyama A Utsumi H Kuwano K Kawakubo T . Necessity of neutrophil-to-lymphocyte ratio monitoring for hypothyroidism using nivolumab in patients with cancer. World J Clin Oncol. (2022) 13:641–51. doi: 10.5306/wjco.v13.i7.641 PMC9346426 36157155

[48] Inoue H Shiozaki A Fujiwara H Konishi H Kiuchi J Ohashi T . Absolute lymphocyte count and C-reactive protein-albumin ratio can predict prognosis and adverse events in patients with recurrent esophageal cancer treated with nivolumab therapy. Oncol Lett. (2022) 24:257. doi: 10.3892/ol.2022.13377 35765281 PMC9219019

[49] Jia X Chu X Jiang L Li Y Zhang Y Mao Z . Predicting checkpoint inhibitors pneumonitis in non-small cell lung cancer using a dynamic online hypertension nomogram. Lung Cancer. (2022) 170:74–84. doi: 10.1016/j.lungcan.2022.06.001 35717705

[50] Lu X Wan J Shi H . Platelet-to-lymphocyte and neutrophil-to-lymphocyte ratios are associated with the efficacy of immunotherapy in stage III/IV non-small cell lung cancer. Oncol Lett. (2022) 24:266. doi: 10.3892/ol.2022.13386 35782904 PMC9247654

[51] Ma Y Ma X Wang J Wu S Wang J Cao B . Absolute eosinophil count may be an optimal peripheral blood marker to identify the risk of immune-related adverse events in advanced Malignant tumors treated with PD-1/PD-L1 inhibitors: a retrospective analysis. World J Surg Oncol. (2022) 20:242. doi: 10.1186/s12957-022-02695-y 35897018 PMC9331074

[52] Matsuo M Yasumatsu R Masuda M Toh S Wakasaki T Hashimoto K . Inflammation-based prognostic score as a prognostic biomarker in patients with recurrent and/or metastatic head and neck squamous cell carcinoma treated with nivolumab therapy. In Vivo. (2022) 36:907–17. doi: 10.21873/invivo.12780 PMC8931916 35241549

[53] Sonehara K Tateishi K Araki T Komatsu M Akahane J Yamamoto H . Predictive factors correlated with the development of immune-related adverse events in patients with non-small cell lung cancer treated with immune checkpoint inhibitors. Cancer Manag Res. (2022) 14:427–35. doi: 10.2147/CMAR.S347852 PMC8818764 35140520

[54] Tada T Kumada T Hiraoka A Hirooka M Kariyama K Tani J . Neutrophil-lymphocyte ratio predicts early outcomes in patients with unresectable hepatocellular carcinoma treated with atezolizumab plus bevacizumab: a multicenter analysis. Eur J Gastroenterol Hepatol Jun 1. (2022) 34:698–706. doi: 10.1097/MEG.0000000000002356 35170529

[55] Takayasu S Mizushiri S Watanuki Y Yamagata S Usutani M Nakada Y . Eosinophil counts can be a predictive marker of immune checkpoint inhibitor-induced secondary adrenal insufficiency: a retrospective cohort study. Sci Rep Jan 25. (2022) 12:1294. doi: 10.1038/s41598-022-05400-x PMC8789805 35079086

[56] Wu S Bai H Zhang L He J Luo X Wang S . Cardiovascular adverse events induced by immune checkpoint inhibitors: A real world study from 2018 to 2022. Front Cardiovasc Med. (2022) 9:969942. doi: 10.3389/fcvm.2022.969942 36035942 PMC9399397

[57] Wu Y Li D Wu M Yang Y Shen M Chen K . Peripheral absolute eosinophil count identifies the risk of serious immune-related adverse events in non-small cell lung cancer. Front Oncol. (2022) 12:1004663. doi: 10.3389/fonc.2022.1004663 36313675 PMC9608122

[58] Wu YL Fulgenzi CAM D’Alessio A Cheon J Nishida N Saeed A . Neutrophil-to-lymphocyte and platelet-to-lymphocyte ratios as prognostic biomarkers in unresectable hepatocellular carcinoma treated with atezolizumab plus bevacizumab. Cancers (Basel). (2022) 14(23):5834. doi: 10.3390/cancers14235834 36497316 PMC9737420

[59] Yu Y Wang S Su N Pan S Tu B Zhao J . Increased circulating levels of CRP and IL-6 and decreased frequencies of T and B lymphocyte subsets are associated with immune-related adverse events during combination therapy with PD-1 inhibitors for liver cancer. Front Oncol. (2022) 12:906824. doi: 10.3389/fonc.2022.906824 35756643 PMC9232255

[60] Zhang Z Xie T Qi C Zhang X Shen L Peng Z . Peripheral blood biomarkers predictive of efficacy outcome and immune-related adverse events in advanced gastrointestinal cancers treated with checkpoint inhibitors. Cancers (Basel). (2022) 14(15):3736. doi: 10.3390/cancers14153736 35954401 PMC9367581

[61] Zhao L Li Y Jiang N Song X Xu J Zhu X . Association of blood biochemical indexes and antibiotic exposure with severe immune-related adverse events in patients with advanced cancers receiving PD-1 inhibitors. J Immunother. May 1. (2022) 45:210–6. doi: 10.1097/CJI.0000000000000415 PMC8986630 35250004

[62] Dharmapuri S Ozbek U Jethra H Jun T Marron TU Saeed A . Baseline neutrophil-lymphocyte ratio and platelet-lymphocyte ratio appear predictive of immune treatment related toxicity in hepatocellular carcinoma. World J Gastrointest Oncol. (2023) 15:1900–12. doi: 10.4251/wjgo.v15.i11.1900 PMC10701235 38077640

[63] Fujimoto A Koutake Y Hisamatsu D Ookubo N Yabuuchi Y Kamimura G . Risk factors indicating immune-related adverse events with combination chemotherapy with immune checkpoint inhibitors and platinum agents in patients with non-small cell lung cancer: a multicenter retrospective study. Cancer Immunol Immunother. (2023) 72:2169–78. doi: 10.1007/s00262-023-03408-4 PMC10992420 36849845

[64] Gao J Zhang P Tang M Nie X Yuan Y Yang F . Predictors of immune checkpoint inhibitor-related adverse events in older patients with lung cancer: a prospective real-world analysis. J Cancer Res Clin Oncol. (2023) 149:8993–9006. doi: 10.1007/s00432-023-04792-1 37160811 PMC11798061

[65] Gao W Liu Q Zhou Y Yang M Yu Y . The predictive model construction for immune-related adverse events in non-small cell lung cancer patients receiving immunotherapy. Technol Cancer Res Treat. (2023) 22:15330338231206705. doi: 10.1177/15330338231206705 37927008 PMC10629333

[66] Haraguchi M Nakao Y Narita S Matsumoto K Fukushima M Sasaki R . Peripheral lymphocyte fluctuation as an indicator of severe immune-related adverse events in patients treated with immune checkpoint inhibitors. Cancer Med. (2023) 12:10636–46. doi: 10.1002/cam4.5816 PMC10225184 36934436

[67] Hu WT Zhang Q Zhang Z He X Zhou M Guo Y . Eosinophil and IFN-gamma associated with immune-related adverse events as prognostic markers in patients with non-small cell lung cancer treated with immunotherapy. Front Immunol. (2023) 14:1112409. doi: 10.3389/fimmu.2023.1112409 36949952 PMC10025375

[68] Mehra T Dongre K Boesing M Frei P Suenderhauf C Zippelius A . Pre-treatment comorbidities, C-reactive protein and eosinophil count, and immune-related adverse events as predictors of survival with checkpoint inhibition for multiple tumour entities. Cancer Med. (2023) 12:12253–62. doi: 10.1002/cam4.5919 PMC10278511 37084178

[69] Pan C Wu QV Voutsinas J Houlton JJ Barber B Futran N . Neutrophil to lymphocyte ratio and peripheral blood biomarkers correlate with survival outcomes but not response among head and neck and salivary cancer treated with pembrolizumab and vorinostat. Head Neck. (2023) 45:391–7. doi: 10.1002/hed.27252 PMC9812876 36412064

[70] Qiu X Shi Z Tong F Lu C Zhu Y Wang Q . Biomarkers for predicting tumor response to PD-1 inhibitors in patients with advanced pancreatic cancer. Hum Vaccin Immunother. (2023) 19:2178791. doi: 10.1080/21645515.2023.2178791 36809234 PMC10026926

[71] Teijeira L Martinez M Moreno A Elejoste I Ibanez-Beroiz B Arrazubi V . Baseline circulating blood cell counts and ratios and changes therein for predicting immune-related adverse events during immune checkpoint inhibitor therapy: A multicenter, prospective, observational, pan-cancer cohort study with a gender perspective. Cancers (Basel). (2023) 16(1):151. doi: 10.3390/cancers16010151 38201577 PMC10778233

[72] Kuwano A Yada M Tanaka K Koga Y Nagasawa S Masumoto A . Neutrophil-to-lymphocyte ratio predicts immune-related adverse events in patients with hepatocellular carcinoma treated with atezolizumab plus bevacizumab. Cancer Diagn Progn. (2024) 4:34–41. doi: 10.21873/cdp.10282 38173658 PMC10758843

[73] Sue M Takeuchi Y Hirata S Takaki A Otsuka M . Impact of nutritional status on neutrophil-to-lymphocyte ratio as a predictor of efficacy and adverse events of immune check-point inhibitors. Cancers (Basel). (2024) 16(10):1811. doi: 10.3390/cancers16101811 38791890 PMC11120021

[74] Wang J Ma Y Lin H Wang J Cao B . Predictive biomarkers for immune-related adverse events in cancer patients treated with immune-checkpoint inhibitors. BMC Immunol. (2024) 25:8. doi: 10.1186/s12865-024-00599-y 38267897 PMC10809515

[75] Yang M Zhang J Wei D Yu T Chen Z Liu X . Inflammatory markers predict survival in patients with postoperative urothelial carcinoma receiving tislelizumab (PD-1 inhibitor) adjuvant therapy. BMC Cancer. (2024) 24:196. doi: 10.1186/s12885-024-11969-5 38347460 PMC10860305

[76] Zhang B Chen J Yu H Li M Cai M Chen L . Prognostic nutritional index predicts efficacy and immune-related adverse events of first-line chemoimmunotherapy in patients with extensive-stage small-cell lung cancer. J Inflammation Res. (2024) 17:1777–88. doi: 10.2147/JIR.S450804 PMC10959246 38523686

[77] Yin Q Wu L Han L Zheng X Tong R Li L . Immune-related adverse events of immune checkpoint inhibitors: a review. Front Immunol. (2023) 14:1167975. doi: 10.3389/fimmu.2023.1167975 37304306 PMC10247998

[78] Yan T Yu L Zhang J Chen Y Fu Y Tang J . Achilles’ Heel of currently approved immune checkpoint inhibitors: immune related adverse events. Front Immunol. (2024) 15:1292122. doi: 10.3389/fimmu.2024.1292122 38410506 PMC10895024

[79] Casagrande S Sopetto GB Bertalot G Bortolotti R Racanelli V Caffo O . Immune-related adverse events due to cancer immunotherapy: immune mechanisms and clinical manifestations. Cancers. (2024) 16(7):1440. doi: 10.3390/cancers16071440 38611115 PMC11011060

[80] Oh DY Cham J Zhang L Fong G Kwek SS Klinger M . Immune toxicities elicted by CTLA-4 blockade in cancer patients are associated with early diversification of the T-cell repertoire. Cancer Res. (2017) 77:1322–30. doi: 10.1158/0008-5472.CAN-16-2324 PMC5398199 28031229

[81] Robert L Tsoi J Wang X Emerson R Homet B Chodon T . CTLA4 blockade broadens the peripheral T-cell receptor repertoire. Clin Cancer Res. (2014) 20:2424–32. doi: 10.1158/1078-0432.CCR-13-2648 PMC4008652 24583799

[82] Berner F Bomze D Diem S Ali OH Fässler M Ring S . Association of checkpoint inhibitor-induced toxic effects with shared cancer and tissue antigens in non-small cell lung cancer. JAMA Oncol. (2019) 5:1043–7. doi: 10.1001/jamaoncol.2019.0402 PMC6487908 31021392

[83] Johnson DB Balko JM Compton ML Chalkias S Gorham J Xu Y . Fulminant myocarditis with combination immune checkpoint blockade. N Engl J Med. (2016) 375:1749–55. doi: 10.1056/NEJMoa1609214 PMC5247797 27806233

[84] Tanaka A Sakaguchi S . Regulatory T cells in cancer immunotherapy. Cell Res. (2017) 27:109–18. doi: 10.1038/cr.2016.151 PMC5223231 27995907

[85] Bennett CL Christie J Ramsdell F Brunkow ME Ferguson PJ Whitesell L . The immune dysregulation, polyendocrinopathy, enteropathy, X-linked syndrome (IPEX) is caused by mutations of FOXP3. Nat Genet. (2001) 27:20–1. doi: 10.1038/83713 11137993

[86] König D Läubli H . Mechanisms of immune-related complications in cancer patients treated with immune checkpoint inhibitors. Pharmacology. (2021) 106:123–36. doi: 10.1159/000509081 32721966

[87] Ludwig RJ Vanhoorelbeke K Leypoldt F Kaya Z Bieber K Mclachlan SM . Mechanisms of autoantibody-induced pathology. Front Immunol. (2017) 8:603. doi: 10.3389/fimmu.2017.00603 28620373 PMC5449453

[88] Osorio JC Ni A Chaft JE Pollina R Kasler MK Stephens D . Antibody-mediated thyroid dysfunction during T-cell checkpoint blockade in patients with non-small-cell lung cancer. Ann Oncol. (2017) 28:583–9. doi: 10.1093/annonc/mdw640 PMC5834017 27998967

[89] Tarhini AA Zahoor H Lin Y Malhotra U Sander C Butterfield LH . Baseline circulating IL-17 predicts toxicity while TGF-β1 and IL-10 are prognostic of relapse in ipilimumab neoadjuvant therapy of melanoma. J Immunother Cancer. (2015) 3:39. doi: 10.1186/s40425-015-0081-1 26380086 PMC4570556

[90] Khan S Gerber DE . Autoimmunity, checkpoint inhibitor therapy and immune-related adverse events: A review. Semin Cancer Biol. (2020) 64:93–101. doi: 10.1016/j.semcancer.2019.06.012 PMC6980444 31330185

[91] Wang Y Liu L . Immunological factors, important players in the development of asthma. BMC Immunol. (2024) 25:50. doi: 10.1186/s12865-024-00644-w 39060923 PMC11282818

[92] Vakkila J Lotze MT . Inflammation and necrosis promote tumour growth. Nat Rev Immunol. (2004) 4:641–8. doi: 10.1038/nri1415 15286730

[93] Carretero R Sektioglu IM Garbi N Salgado OC Beckhove P Hämmerling GJ . Eosinophils orchestrate cancer rejection by normalizing tumor vessels and enhancing infiltration of CD8(+) T cells. Nat Immunol. (2015) 16:609–17. doi: 10.1038/ni.3159 25915731

[94] Lucarini V Ziccheddu G Macchia I La Sorsa V Peschiaroli F Buccione C . IL-33 restricts tumor growth and inhibits pulmonary metastasis in melanoma-bearing mice through eosinophils. Oncoimmunology. (2017) 6:e1317420. doi: 10.1080/2162402X.2017.1317420 28680750 PMC5486175

[95] Weide B Martens A Hassel JC Berking C Postow MA Bisschop K . Baseline biomarkers for outcome of melanoma patients treated with pembrolizumab. Clin Cancer Res. (2016) 22:5487–96. doi: 10.1158/1078-0432.CCR-16-0127 PMC5572569 27185375

[96] Gebhardt C Sevko A Jiang H Lichtenberger R Reith M Tarnanidis K . Myeloid cells and related chronic inflammatory factors as novel predictive markers in melanoma treatment with ipilimumab. Clin Cancer Res. (2015) 21:5453–9. doi: 10.1158/1078-0432.CCR-15-0676 26289067

[97] Weinmann SC Pisetsky DS . Mechanisms of immune-related adverse events during the treatment of cancer with immune checkpoint inhibitors. Rheumatol (Oxford). (2019) 58:vii59–67. doi: 10.1093/rheumatology/kez308 PMC6900913 31816080

[98] Woerly G Roger N Loiseau S Capron M . Expression of Th1 and Th2 immunoregulatory cytokines by human eosinophils. Int Arch Allergy Immunol. (1999) 118:95–7. doi: 10.1159/000024038 10224349

[99] Kuo AM-S Gu S Stoll J Moy AP Dusza SW Gordon A . Management of immune-related cutaneous adverse events with dupilumab. J Immunother Cancer. (2023) 11(6):e007324. doi: 10.1136/jitc-2023-007324 37270183 PMC10255229

[100] Larsen BT Chae JM Dixit AS Hartman TE Peikert T Roden AC . Clinical and histopathologic features of immune checkpoint inhibitor-related pneumonitis. Am J Surg Pathol. (2019) 43:1331–40. doi: 10.1097/PAS.0000000000001298 31162288

[101] Nakamura M Otsuka T Kumagai M Arisawa T . Pembrolizumab-induced eosinophilic gastrointestinal disorders. Intern Med. (2022) 61:267–9. doi: 10.2169/internalmedicine.7725-21 PMC8851178 34248122

[102] Yanagibashi T Satoh M Nagai Y Koike M Takatsu K . Allergic diseases: From bench to clinic - Contribution of the discovery of interleukin-5. Cytokine. (2017) 98:59–70. doi: 10.1016/j.cyto.2016.11.011 28863833

[103] Zhu S Fu Y Zhu B Zhang B Wang J . Pneumonitis induced by immune checkpoint inhibitors: from clinical data to translational investigation. Front In Oncol. (2020) 10:1785. doi: 10.3389/fonc.2020.01785 PMC7518129 33042827

[104] Nøst TH Alcala K Urbarova I Byrne KS Guida F Sandanger TM . Systemic inflammation markers and cancer incidence in the UK Biobank. Eur J Epidemiol. (2021) 36:841–8. doi: 10.1007/s10654-021-00752-6 PMC8416852 34036468

[105] Masucci MT Minopoli M Carriero MV . Tumor associated neutrophils. Their role in tumorigenesis, metastasis, prognosis and therapy. Front In Oncol. (2019) 9:1146. doi: 10.3389/fonc.2019.01146 PMC6874146 31799175

[106] Swierczak A Mouchemore KA Hamilton JA Anderson RL . Neutrophils: important contributors to tumor progression and metastasis. Cancer Metastasis Rev. (2015) 34:735–51. doi: 10.1007/s10555-015-9594-9 26361774

[107] Dib PRB Quirino-Teixeira AC Merij LB Pinheiro MBM Rozini SV Andrade FB . Innate immune receptors in platelets and platelet-leukocyte interactions. J Leukoc Biol. (2020) 108:1157–82. doi: 10.1002/JLB.4MR0620-701R 32779243

[108] Rolfes V Ribeiro LS Hawwari I Böttcher L Rosero N Maasewerd S . Platelets fuel the inflammasome activation of innate immune cells. Cell Rep. (2020) 31:107615. doi: 10.1016/j.celrep.2020.107615 32402278 PMC7225754

[109] Repsold L Joubert AM . Platelet function, role in thrombosis, inflammation, and consequences in chronic myeloproliferative disorders. Cells. (2021) 10(11):3034. doi: 10.3390/cells10113034 34831257 PMC8616365

[110] Olsson AK Cedervall J . The pro-inflammatory role of platelets in cancer. Platelets. (2018) 29:569–73. doi: 10.1080/09537104.2018.1453059 29584534

[111] Kurtul A Ornek E . Platelet to lymphocyte ratio in cardiovascular diseases: a systematic review. Angiology. (2019) 70:802–18. doi: 10.1177/0003319719845186 31030530

[112] Cho KI Ann SH Singh GB Her A-Y Shin E-S . Combined usefulness of the platelet-to-lymphocyte ratio and the neutrophil-to-lymphocyte ratio in predicting the long-term adverse events in patients who have undergone percutaneous coronary intervention with a drug-eluting stent. PloS One. (2015) 10:e0133934. doi: 10.1371/journal.pone.0133934 26207383 PMC4514869

[113] Bai R Li L Chen X Chen N Song W Zhang Y . Correlation of peripheral blood parameters and immune-related adverse events with the efficacy of immune checkpoint inhibitors. J Oncol. (2021) 2021:9935076. doi: 10.1155/2021/9935076 34335763 PMC8292079

[114] Ferrari F Giannini A . Approaches to prevention of gynecological Malignancies. BMC Womens Health. (2024) 24:254. doi: 10.1186/s12905-024-03100-4 38654319 PMC11036672

[115] Giannini A Di Donato V Schiavi MC May J Panici PB Congiu MA . Predictors of postoperative overall and severe complications after surgical treatment for endometrial cancer: The role of the fragility index. Int J Gynaecol Obstet. (2020) 148:174–80. doi: 10.1002/ijgo.13020 31657456

[116] Di Donato V Di Pinto A Giannini A Caruso G D'oria O Tomao F . Modified fragility index and surgical complexity score are able to predict postoperative morbidity and mortality after cytoreductive surgery for advanced ovarian cancer. Gynecol Oncol. (2021) 161(1):4–10. doi: 10.1016/j.ygyno.2020.08.022 33223220

