# Supplementary material for: Peripheral blood cell counts as predictors of immune-related adverse events in cancer patients receiving immune checkpoint inhibitors: a systematic review and meta-analysis
Source: Front Immunol. 2025 Jan 30;16:1528084. doi: 10.3389/fimmu.2025.1528084 (PMC11821924; doi:10.3389/fimmu.2025.1528084)
Supplement: Supplementary file 1 [file DataSheet1.zip › Supplementary material/Supplementary Table 1.docx]

Supplementary Table 1 Search strategy of including studies

| Search strategy (example as Ovid Medline) |
| --- |
| 1 Immune Checkpoint Inhibitors/  2 immune checkpoint inhibitor.mp.  3 immune checkpoint inhibitors.mp.  4 immune checkpoint blockade.mp.  5 immune checkpoint therapy.mp.  6 immunotherapy.mp.  7 immune related adverse events.mp.  8 immune related adverse event.mp.  9 or/1-8  10 CTLA-4.mp.  11 CTLA4.mp.  12 cytotoxic T-lymphocyte-associated protein 4.mp.  13 CD152.mp.  14 PD-1.mp.  15 PD1.mp.  16 programmed cell death protein 1.mp.  17 CD279.mp.  18 PD-L1.mp.  19 PDL1.mp.  20 programmed death-ligand 1.mp.  21 CD274.mp.  22 Pembrolizumab.mp.  23 Nivolumab.mp.  24 Cemiplimab.mp.  25 Tislelizumab.mp.  26 Sintilimab.mp.  27 Atezolizumab.mp.  28 Durvalumab.mp.  29 Avelumab.mp.  30 Ipilimumab.mp.  31 Tremelimumab.mp.  32 or/10-31  33 9 or 32  34 Neutrophils/  35 neutrophil.mp.  36 neutrophils.mp.  37 ANC.mp.  38 or/34-37  39 Lymphocytes/  40 lymphocyte.mp.  41 lymphocytes.mp.  42 lymphoid cell.mp.  43 lymphoid cells.mp.  44 ALC.mp.  45 or/39-44  46 Monocytes/  47 monocyte.mp.  48 monocytes.mp.  49 AMC.mp.  50 or/46-49  51 Eosinophils/  52 eosinophil.mp.  53 eosinophils.mp.  54 AEC.mp.  55 or/51-54  56 Blood Platelets/  57 blood platelet.mp.  58 blood platelets.mp.  59 platelet.mp.  60 platelets.mp.  61 thrombocyte.mp.  62 thrombocytes.mp.  63 PLT.mp.  64 or/56-63  65 NLR.mp.  66 neutrophil to lymphocyte ratio.mp.  67 neutrophil-lymphocyte ratio.mp.  68 or/65-67  69 PLR.mp.  70 platelet to lymphocyte ratio.mp.  71 platelet-lymphocyte ratio.mp.  72 or/69-71  73 MLR.mp.  74 monocyte to lymphocyte ratio.mp.  75 monocyte-lymphocyte ratio.mp.  76 or/73-75  77 LMR.mp.  78 lymphocyte to monocyte ratio.mp.  79 lymphocyte-monocyte ratio.mp.  80 or/77-79  81 38 or 45 or 50 or 55 or 64 or 68 or 72 or 76 or 80  82 Risk Factors/  83 risk factor*.mp.  84 predicto*.mp.  85 exposur*.mp.  86 risk.mp.  87 or/82-86  88 33 and 81 and 87 |
