# Supplementary material for: Peripheral blood cell counts as predictors of immune-related adverse events in cancer patients receiving immune checkpoint inhibitors: a systematic review and meta-analysis
Source: Front Immunol. 2025 Jan 30;16:1528084. doi: 10.3389/fimmu.2025.1528084 (PMC11821924; doi:10.3389/fimmu.2025.1528084)
Supplement: Supplementary file 1 [file DataSheet1.zip › Supplementary material/Supplementary Table 2.docx]

Supplementary Table 2 General data of the included studies

| **Author** | **Published year** | **irAE type** | **Total** | | | **AE group** | | | **Without AE group** | | |
| --- | --- | --- | --- | --- | --- | --- | --- | --- | --- | --- | --- |
|  |  |  | total | male | age | sample | male | age | sample | male | age |
| **Dwight H Owen** | 2018 | All types of irAE | 91 | 39 | 67 (40-87) | 27 | 14 | NG | 64 | 25 | NG |
| **Yoshiyuki Nakamura** | 2019 | All types of irAE | 45 | 25 | 69.3 (42-85) | 26 | NG | NG | 19 | NG | NG |
| **Alberto Pavan** | 2019 | All types of irAE | 184 | 125 | 67 (37–83) | 60 | NG | NG | 124 | NG | NG |
| **Yu Nakanishi** | 2019 | Interstitial lung disease | 83 | 58 | 68 (34-85) | 14 | 10 | 65 (49-80) | 69 | 48 | 68 (34-85) |
| **Jun Fukihara** | 2019 | Pneumonitis | 170 | NG | NG | 27 | 20 | 67 (58-73) | 143 | 105 | 70 (63-73) |
| **Yeonghee Eun** | 2019 | All types of irAE | 391 | NG | NG | 67 | 45 | 59 (52–66) | 324 | 202 | 60 (52–69) |
| **Koichiro Ogihara** | 2020 | ir-SAE | 78 | NG | NG | 19 | NG | NG | 59 | NG | NG |
| **Lihong Peng** | 2020 | All types of irAE | 102 | 87 | NG | 39 | NG | NG | 63 | NG | NG |
| **Shilpa Grover** | 2020 | Colitis | 213 | NG | NG | 37 | 22 | NG | 176 | 102 | NG |
| **Kazuo Kobayashi** | 2020 | All types of irAE | 53 | 40 | 67 (41-85) | 24 | NG | NG | 29 | NG | NG |
| **Ganessan Kichenadasse** | 2020 | All types of irAE | 1548 | 936 | 64 (57–70) | 340 | 211 | 66 (58–72) | 1124 | 673 | 63 (56–70) |
| **Xiangling Chu** | 2020 | Pneumonitis | 300 | 240 | 60.5(20-82) | 54 | 48 | NG | 246 | 192 | NG |
| **Zsofia D Drobni** | 2020 | Myocarditis | 110 | NG | NG | 55 | 41 | 67±15 | 55 | 30 | 66±16 |
| **Melissa Y Y Moey** | 2020 | Major adverse  cardiac events | 196 | NG | NG | 23 | 15 | 68.7±1.8 | 173 | 99 | 64.3±0.8 |
| **Ryosuke Matsukane** | 2021 | All types of irAE | 275 | 203 | 68 (16–89) | 121 | NG | NG | 154 | NG | NG |
| **Eduard Roussel** | 2021 | All types of irAE | 113 | 46 | 66 (59-73) | 22 | NG | NG | 91 | NG | NG |
| **Xiaona Fan** | 2021 | All types of irAE | 111 | 56 | NG | 30 | NG | NG | 81 | NG | NG |
| **Pei Yi Lee** | 2021 | All types of irAE | 147 | NG | NG | 91 | 60 | 61 (52–70) | 56 | 39 | 63 (56–68) |
| **Despina Michailidou** | 2021 | All types of irAE | 470 | 275 | 65 (56–71) | 156 | NG | NG | 314 | NG | NG |
| **Ashish Manne** | 2021 | All types of irAE | 160 | 79 | 64(17-93) | 46 | 25 | 65±9.36 | 114 | 54 | 62.5±13.6 |
| **Airi Fujimoto** | 2021 | All types of irAE | 115 | NG | NG | 45 | 32 | 68 (45–87) | 70 | 52 | 69 (44–85) |
| **Rilan Bai** | 2021 | All types of irAE | 105 | 81 | 61(24-84) | 41 | NG | NG | 64 | NG | NG |
| **Lea Daniello** | 2021 | All types of irAE | 894 | NG | NG | 198 | 117 | 65±12 | 696 | 419 | 65±12 |
| **Dan-Yun Ruan** | 2021 | All types of irAE | 58 | 41 | 60 (52–66) | 14 | NG | NG | 44 | NG | NG |
| **Yuequan Shi** | 2021 | All types of irAE | 103 | 68 | 66 (61-71) | 38 | NG | 67 (61-72) | 65 | NG | 65 (61-71) |
| **Shinobu Takayasu** | 2022 | Adrenal insufficiency | 525 | 394 | 67.1±10.0 | 19 | NG | NG | 506 | NG | NG |
| **Kei Sonehara** | 2022 | All types of irAE | 113 | 91 | 70 (29-87) | 44 | 38 | 71 (29–87) | 69 | 56 | 68 (42–83) |
| **Toshifumi Tada** | 2022 | All types of irAE | 249 | 211 | 73 (68-79) | 148 | NG | NG | 101 | NG | NG |
| **Mioko Matsuo** | 2022 | All types of irAE | 164 | 127 | 65 (23-87) | 52 | NG | NG | 112 | NG | NG |
| **Lijun Zhao** | 2022 | ir-SAE | 168 | NG | 60.8±10.2 | 42 | 33 | 60.3±9.6 | 236 | 99 | 60.9±10.4 |
| **Manuel Sánchez**  **Cánovas** | 2022 | Thrombosis | 665 | 463 | NG | 56 | NG | NG | 609 | NG | NG |
| **Xue Chen** | 2022 | Cardiotoxicity | 1101 | NG | NG | 73 | 56 | 59.8±11.7 | 974 | 657 | 59.5±11.2 |
| **Xiaohui Jia** | 2022 | Pneumonitis | 418 | NG | 61(27-78) | 88 | NG | NG | 330 | NG | NG |
| **Yingying Yu** | 2022 | All types of irAE | 67 | 57 | 57.2±9.7 | 36 | 33 | 57.9±9.7 | 31 | 24 | 56.4±9.9 |
| **Afaf Abed** | 2022 | All types of irAE | 179 | 101 | NG | 77 | NG | NG | 102 | NG | NG |
| **Hiroyuki Inoue** | 2022 | All types of irAE | 41 | 34 | 68(51–81) | 24 | NG | NG | 17 | NG | NG |
| **Xiaojuan Lu** | 2022 | All types of irAE | 133 | 56 | NG | 22 | NG | NG | 111 | NG | NG |
| **Yan Ma** | 2022 | All types of irAE | 95 | 66 | 62 (30-80) | 53 | NG | NG | 42 | NG | NG |
| **Zhening Zhang** | 2022 | All types of irAE | 234 | 172 | NG | 139 | NG | NG | 95 | NG | NG |
| **Si Wu** | 2022 | Cardiovascular  adverse events | 495 | NG | NG | 64 | 52 | 61.8±10.0 | 431 | 317 | 62.3±10.2 |
| **Ako Gannichida** | 2022 | Hypothyroidism | 104 | 69 | 69 (32-91) | 21 | 14 | 70 (45-91) | 83 | 55 | 68 (32-88) |
| **Cho-Han Chiang** | 2022 | Cardiotoxicity | 868 | 556 | 69(59–77) | 67 | NG | NG | 801 | NG | NG |
| **Zhiyao Bao** | 2022 | Renal | 328 | 264 | 63.27±9.36 | 41 | 34 | 63.28±8.87 | 286 | 230 | 63.28±8.87 |
| **Yan Wu** | 2022 | All types of irAE | 213 | 181 | (28-90) | 122 | 102 | NG | 91 | 79 | NG |
| **Yue Linda Wu** | 2022 | All types of irAE | 296 | 245 | 66 (59–73) | 63 | NG | NG | 220 | NG | NG |
| **Cassie Pan** | 2023 | ir-SAE | 50 | NG | NG | NG | NG | NG | NG | NG | NG |
| **Xin Qiu** | 2023 | All types of irAE | 67 | 44 | 61.4 (33–84) | 50 | NG | NG | 17 | NG | NG |
| **Airi Fujimoto** | 2023 | All types of irAE | 315 | NG | NG | 50 | 41 | 69 (40–80) | 265 | 199 | 68 (42–86) |
| **Masafumi Haraguchi** | 2023 | All types of irAE | 196 | 146 | NG | 69 | 54 | NG | 127 | 92 | 71 (24–93) |
| **Wei-Ting Hu** | 2023 | All types of irAE | 149 | 116 | NG | 83 | 70 | 67±9 | 66 | 46 | 62±11 |
| **Tarun Mehra** | 2023 | All types of irAE | 229 | 154 | 65 (18–91) | 75 | NG | NG | 154 | NG | NG |
| **Jiayi Gao** | 2023 | All types of irAE | 201 | 169 | 71(65–92) | 98 | NG | NG | 103 | NG | NG |
| **Weitong Gao** | 2023 | All types of irAE | 484 | 347 | NG | 81 | NG | NG | 403 | NG | NG |
| **Sirish Dharmapuri** | 2023 | All types of irAE | 361 | NG | NG | 167 | NG | NG | 194 | NG | NG |
| **Lucía Teijeira** | 2023 | All types of irAE | 145 | 112 | 65.8±9.7 | 52 | NG | NG | 93 | NG | NG |
| **Akifumi Kuwano** | 2024 | All types of irAE | 69 | 57 | 73(65.5-79.5) | 12 | 10 | 76.5(71.5-81.8) | 57 | 47 | 72(64.0-78.5) |
| **Jingting Wang** | 2024 | All types of irAE | 418 | 298 | 64.1(21-87) | 176 | NG | NG | 242 | NG | NG |
| **Meng Yang** | 2024 | All types of irAE | 133 | 95 | 64.6±11.0 | 46 | NG | NG | 87 | NG | NG |
| **Baishen Zhang** | 2024 | All types of irAE | 114 | 102 | NG | 53 | NG | NG | 61 | NG | NG |
| **Masahiko Sue** | 2024 | All types of irAE | 1234 | 892 | 69 (60–95) | 333 | NG | NG | 901 | NG | NG |
