# Supplementary material for: Peripheral blood cell counts as predictors of immune-related adverse events in cancer patients receiving immune checkpoint inhibitors: a systematic review and meta-analysis
Source: Front Immunol. 2025 Jan 30;16:1528084. doi: 10.3389/fimmu.2025.1528084 (PMC11821924; doi:10.3389/fimmu.2025.1528084)
Supplement: Supplementary file 1 [file DataSheet1.zip › Supplementary material/Supplementary Table 3.docx]

Supplementary Table 3 The summary of the different types of irAE in the including studies

| **Author** | **Published year** | **Immune checkpoint**  **inhibitors** | **total sample** | **Dermatologic disorders** | **Pneumonitis** | **Musculoskeletal disorders** | **Endocrinopathy** | **Gastrointestinal disorders** | **Liver injury** | **Rheumatic injury** | **Cardiovascular injury** |
| --- | --- | --- | --- | --- | --- | --- | --- | --- | --- | --- | --- |
| **Dwight H Owen** | 2018 | Nivolumab, Pembrolizumab, Atezolizumab | 27* | 6 | 9 | NG | 7 | 3 | 1 | NG | NG |
| **Yoshiyuki Nakamura** | 2019 | Nivolumab, Pembrolizumab | 56 | 15 | 1 | NG | 14 | 6 | 2 | 1 | NG |
| **Alberto Pavan** | 2019 | Nivolumab, Pembrolizumab, Atezolizumab | 60* | 11 | 13 | 5 | 6 | 12 | 12 | NG | NG |
| **Yeonghee Eun** | 2019 | Pembrolizumab | 88 | 49 | 11 | 12 | 7 | 5 | 3 | NG | NG |
| **Lihong Peng** | 2020 | Nivolumab, Pembrolizumab,  Toripalimab, Sintilimab | 39^*^ | 13 | NG | NG | 7 | NG | 9 | NG | NG |
| **Kazuo Kobayashi** | 2020 | Nivolumab | 24* | 9 | 3 | NG | NG | 7 | 1 | NG | NG |
| **Ganessan Kichenadasse** | 2020 | Atezolizumab | 1124 | 306 | 45 | NG | 85 | 15 | 4 | 8 | NG |
| **Ryosuke Matsukane** | 2021 | Nivolumab, Pembrolizumab | 166 | 61 | 26 | NG | 17 | 9 | 5 | NG | 2 |
| **Pei Yi Lee** | 2021 | Nivolumab, Pembrolizumab,Atezolizumab, Avelumab,  Durvalumab, Tremelimumab | 91* | 8 | 5 | NG | 12 | 9 | 11 | 3 | NG |
| **Despina Michailidou** | 2021 | Nivolumab, Pembrolizumab,Cemiplimab, Atezolizumab, Durvalumab, Avelumab,  Ipilimumab, Tremelimumab | 212 | 13 | 25 | NG | 72 | 58 | 18 | 31 | 1 |
| **Airi Fujimoto** | 2021 | Nivolumab, Pembrolizumab,Atezolizumab | 45^*^ | 14 | 8 | NG | 16 | 5 | 2 | NG | 2 |
| **Rilan Bai** | 2021 | Nivolumab, Pembrolizumab,Toripalimab, Sintilimab,  Tislelizumab, Camrelizumab,Atezolizumab, Ipilimumab | 77 | 14 | 3 | NG | 19 | 2 | 13 | NG | 1 |
| **Lea Daniello** | 2021 | Nivolumab, Pembrolizumab,Atezolizumab, Durvalumab | 232 | 23 | 40 | 38 | 44 | 37 | 33 | NG | 4 |
| **Yuequan Shi** | 2021 | Not specified, including  anti–PD-1, anti-PD-L1,  anti-CTLA4 inhibitors | 57 | 16 | 4 | 1 | 12 | 14 | 8 | NG | 3 |
| **Kei Sonehara** | 2022 | Nivolumab, Pembrolizumab,Atezolizumab | 44* | 8 | 8 | 1 | 16 | NG | 5 | NG | NG |
| **Yingying Yu** | 2022 | Nivolumab, Camrelizumab, Sintilimab | 76 | 13 | 1 | NG | 11 | 9 | 1 | 1 | 3 |
| **Afaf Abed** | 2022 | Nivolumab, Pembrolizumab,Atezolizumab | 156 | 34 | 15 | 20 | 12 | 7 | 10 | NG | NG |
| **Hiroyuki Inoue** | 2022 | Nivolumab | 24* | 24 | 3 | NG | 5 | 7 | NG | NG | NG |
| **Yan Ma** | 2022 | Nivolumab, Atezolizumab,  Sintilimab, Camrelizumab | 53* | 11 | 7 | NG | 4 | 4 | 11 | NG | 5 |
| **Zhening Zhang** | 2022 | Nivolumab, Pembrolizumab,Zimberelimab,  Camrelizumab, Sintilimab,  Tislelizumab, Toripalimab,  Atezolizumab,  Sugemalimab, Envafolimab, Nivolumab, Ipilimumab,  Cadolinimab | 260 | 52 | 9 | NG | 49 | 21 | 59 | 35 | 6 |
| **Yan Wu** | 2022 | Not specified, including  anti–PD-1, anti-PD-L1  inhibitor | 122* | 43 | 25 | NG | 40 | 10 | 22 | NG | 6 |
| **Xin Qiu** | 2023 | Pembrolizumab, Sintilimab, Toripalimab | 17* | 7 | 1 | 3 | 13 | NG | 2 | NG | NG |
| **Airi Fujimoto** | 2023 | Nivolumab, Pembrolizumab,Ipilimumab, Atezolizumab | 50* | 16 | 11 | 2 | 12 | 5 | 5 | 1 | NG |
| **Masafumi Haraguchi** | 2023 | Nivolumab, Pembrolizumab,Atezolizumab, Ipilimumab | 99 | 8 | 21 | 2 | 20 | 13 | 35 | NG | NG |
| **Wei-Ting Hu** | 2023 | Not specified, including  anti–PD-1 inhibitor | 128 | 34 | 37 | NG | 10 | 9 | 11 | NG | 11 |
| **Tarun Mehra** | 2023 | Nivolumab, Pembrolizumab,Atezolizumab, Ipilimumab | 137 | 43 | 12 | NG | 26 | 26 | 11 | 15 | NG |
| **Jiayi Gao** | 2023 | Not specified, including  anti–PD-1, anti-PD-L1  inhibitor | 98* | 39 | 40 | 6 | 15 | 4 | 4 | NG | 3 |
| **Weitong Gao** | 2023 | Nivolumab, Pembrolizumab,Camrelizumab, Sintilimab,  Tislelizumab, Toripalimab,  Atezolizumab, Durvalumab,  Ipilimumab | 112 | 20 | 24 | NG | 38 | NG | NG | NG | 19 |
| **Sirish Dharmapuri** | 2023 | Not specified, including  anti–PD-1, anti-CTLA4  inhibitors | 167* | 58 | 19 | NG | 29 | 29 | 69 | 4 | NG |
| **Lucía Teijeira** | 2023 | Nivolumab, Pembrolizumab,Cemiplimab, Atezolizumab, Durvalumab, Avelumab | 52* | 17 | 1 | 2 | 14 | 4 | 2 | 3 | NG |
| **Akifumi Kuwano** | 2024 | Atezolizumab | 12* | NG | 2 | NG | 5 | 1 | NG | NG | NG |
| **Jingting Wang** | 2024 | Nivolumab, Pembrolizumab,Camrelizumab, Sintilimab,  Atezolizumab, Durvalumab | 176* | 29 | 38 | 1 | 43 | 5 | 17 | NG | 34 |
| **Meng Yang** | 2024 | Tislelizumab | 46* | 11 | NG | NG | 3 | 27 | NG | NG | NG |
| **Baishen Zhang** | 2024 | Atezolizumab, Durvalumab | 114 | 14 | 10 | NG | 31 | NG | 10 | NG | NG |
| **Masahiko Sue** | 2024 | Nivolumab, Pembrolizumab,Atezolizumab, Durvalumab, Avelumab, Ipilimumab | 413 | 54 | 71 | 3 | 98 | 34 | 84 | 9 | NG |

Abbreviation: NG: not given, * patient number instead of irAE number
