# Supplementary figures and images for: Peripheral blood cell counts as predictors of immune-related adverse events in cancer patients receiving immune checkpoint inhibitors: a systematic review and meta-analysis
Source: Front Immunol. 2025 Jan 30;16:1528084. doi: 10.3389/fimmu.2025.1528084 (PMC11821924; doi:10.3389/fimmu.2025.1528084)

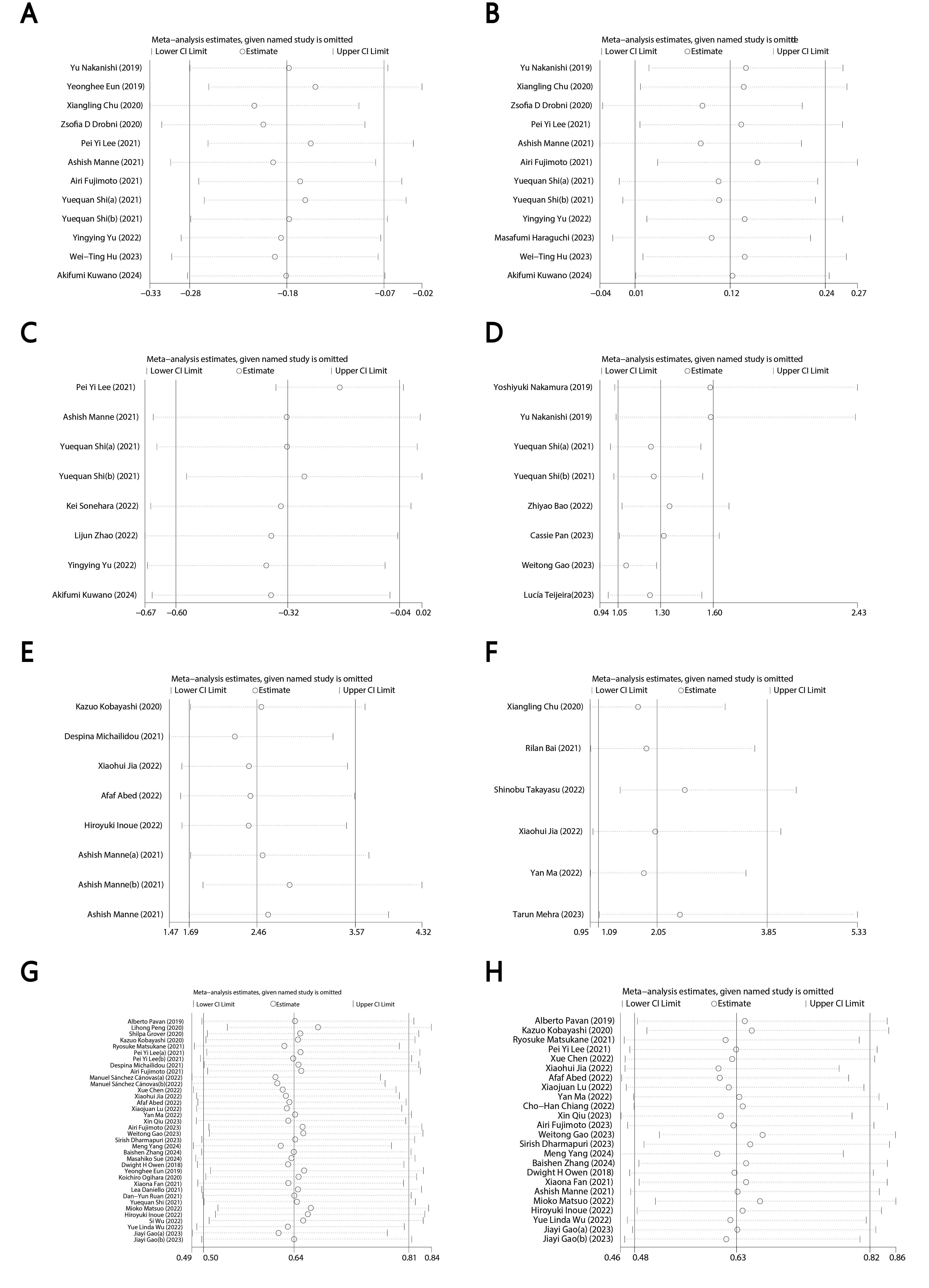

Supplement: Supplementary file 1 [file DataSheet1.zip › Supplementary material/Supplementary Figure1.tif]
